# Computational study of parameter sensitivity in DevR regulated gene expression

**DOI:** 10.1371/journal.pone.0228967

**Published:** 2020-02-13

**Authors:** Jagannath Das, Tarunendu Mapder, Sudip Chattopadhyay, Suman K. Banik

**Affiliations:** 1 Department of Chemistry, Indian Institute of Engineering Science and Technology, Shibpur, Howrah, India; 2 Department of Chemistry, Bose Institute, Kolkata, India; Jackson Laboratory, UNITED STATES

## Abstract

The DevRS two-component system plays a pivotal role in signal transmission and downstream gene regulation in *Mycobacterium tuberculosis*. Under the hypoxic condition, phosphorylated DevR interacts with multiple binding sites at the promoter region of the target genes. In the present work, we carried out a detailed computational analysis to figure out the sensitivity of the kinetic parameters. The set of kinetic parameters takes care of the interaction among phosphorylated DevR and the binding sites, transcription and translation processes. We employ the method of stochastic optimization to quantitate the relevant kinetic parameter set necessary for DevR regulated gene expression. Measures of different correlation coefficients provide the relative ordering of kinetic parameters involved in gene regulation. Results obtained from correlation coefficients are further corroborated by sensitivity amplification.

## Introduction

Tuberculosis is the second most infectious disease in today’s world and is caused by the human pathogen *Mycobacterium tuberculosis* [1]. This highly studied pathogen kills around two million people each year. It is believed that approximately one-third of the world population carries *M. tuberculosis* bacteria within the human body in the inactive state, *viz*. dormant state. Different kinds of environmental and chemical factors trigger its activation. In the development of mycobacterial dormancy and latent tuberculosis, the two-component systems (TCS) plays a pivotal role. Here, it is relevant to note that the TCS is the most important signal transduction pathway in bacteria [2–4]. It is reported that in *M. tuberculosis* there are 11 well defined TCS [5]. The most studied among these TCSs is DevRS and is responsible for the dormancy of *M. tuberculosis* in the host. Analogous to the other TCS, DevRS contains a membrane-bound sensor kinase DevS and a cytoplasmic response regulator DevR. The sensor protein DevS utilizes adenosine triphosphate (ATP) to autophosphorylate a conserved histidine residue under hypoxic, nitric oxide, carbon monoxide, ascorbic acid environment or nutrient starvation conditions. The high energy phosphoryl group is then transferred to the conserved aspartate residue in DevR, the response regulator. Phosphorylated DevR (*R*_*P*_) regulates expression of ∼48 genes along with its operon. Several of these genes contain 20 bp palindromic sequence in the upstream region, known as Dev box, to which *R*_*P*_ binds [6].

**Fig 1:**
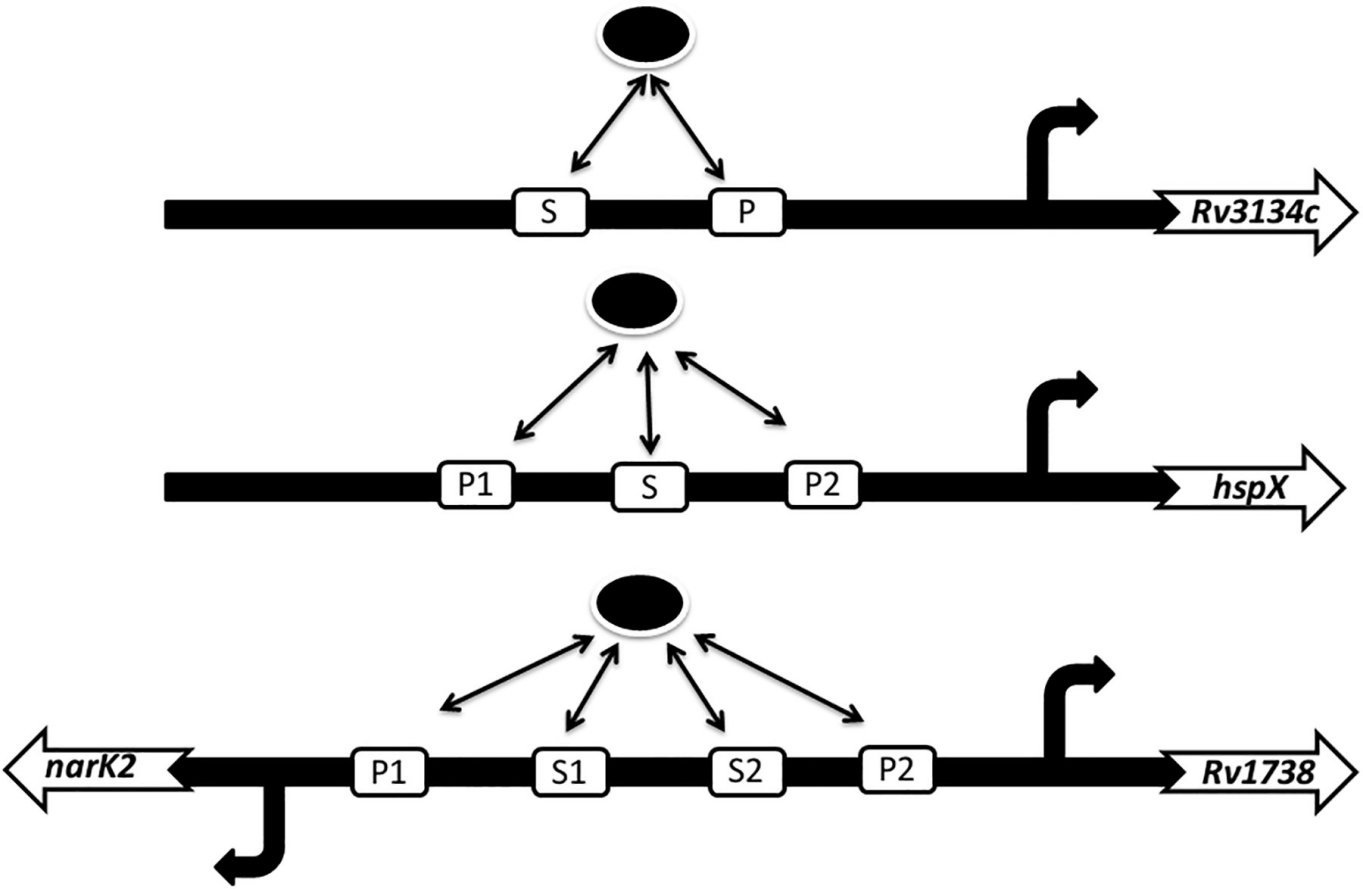
DevR-promoter interaction. Schematic diagram for interaction of phosphorylated DevR (black oval) with different binding sites of the target promoters. Promoters of *Rv3134c* and *hspx* contain two and three binding sites, respectively. A single promoter with four binding sites is shared by *narK2* and *Rv1738*. S (S1 and S2) and P (P1 and P2) stand for secondary and primary binding site, respectively [6].

In the present work, we undertake a computational approach to study interactions between *R*_*P*_ and four of its target genes, e.g., *Rv3134c*, *hspX*, *narK2* and *Rv1738*. A recent report by Chauhan et al [6] provides detailed information about the biochemical interactions between *R*_*P*_ and the four target genes. Based on the affinity of the binding strength of *R*_*P*_ to the binding sites present in the promoter region, the binding sites are broadly classified into two different classes, primary and secondary (see Fig 1). The main objectives of the present communication are two-fold. First, using simulated annealing, a stochastic optimization technique, we optimize the kinetic rate parameters. The optimized parameter set is then used to generate novel experimental profiles [7]. Second, we carried out a sensitivity analysis to figure out the sensitivity of the kinetic parameters related to the binding/unbinding constants, the rate of mRNA production from the promoter-GFP construct and rate of GFP production. Based on the correlation coefficient between the kinetic parameters and the output (GFP), we provide a detailed ordering of the parameters related to the expression of four target genes shown in Fig 1. The analysis based on correlation data sheds light on the complex interaction between DevR and the binding sites and shows how the binding sites are responsible for the gene expression. The sensitivity of the kinetic parameters is then further verified using the measure of sensitivity amplification.

Before proceeding further, we discuss here some of the theoretical developments related to the role of kinetic parameters in gene expression. Recently a computational method, sRACIPE, has been developed to implement stochastic analysis in random circuit perturbation method (RACIPE) [8]. sRACIPE takes care of noisy gene expression along with the parametric variation of a gene regulatory circuit while considering only its topology. The method developed is useful for studying multi-stable biological processes that exhibit fluctuations induced cell-to-cell variation in a cellular population. Implementation of global sensitivity analysis has been addressed earlier to engineer artificial genetic circuits [9] where the authors made an estimation of circuit properties in terms of model parameters, without prior knowledge of precise parameter values. Role of parameter robustness has also been investigated in neurogenic network [10]. A Monte Carlo based computation tool has been developed to identify regions of parameter space that can generate multi-stable states while taking into account fluctuations in parameter space and initial conditions [11].

## Model and methods

### Kinetic model

Based on the available experimental information [6] on interactions of DevR with the promoters of *Rv3134c*, *hspX*, *narK2* and *Rv1738* we employ the kinetic model [12]. The generalized kinetic scheme for phosphorylated DevR (*R*_*P*_) regulated gene expression can be written as

$$\begin{array}{ccc} P_{i}+R_{p} & \overset{k_{bi}}{\underset{k_{ui}}{⇌}} & P_{i}^{*},\\ P_{i}^{*} & \overset{k_{smi}}{⟶} & P_{i}^{*}+mGFP,\\ P_{i}^{*}\ldots P_{N}^{*} & \overset{k_{smc}}{⟶} & P_{i}^{*}\ldots P_{N}^{*}+mGFP,\\ mGFP & \overset{k_{d,m}}{⟶} & \phi ,\\ mGFP & \overset{k_{sg}}{⟶} & mGFP+GFP\\ GFP & \overset{k_{dg}}{⟶} & \phi . \end{array}$$

The above kinetic scheme is valid for a promoter site with *N* numbers of binding sites (*i* = 1…*N*). *P*_*i*_ and $P_{i}^{*}$ stands for the inactive and active state of the promoter, respectively. It is relevant to note that, in our model, each of the *P*_*i*_-s represent the primary and/or secondary binding sites. *mGFP* and *GFP* are for mRNA and GFP, respectively. The kinetic scheme mentioned above needs to be translated into sets of coupled ordinary differential equations (ODEs) to investigate the steady state and temporal dynamics. For detailed kinetic rate equations with associated parameters, we refer to S1 Text and S1 Table.

### Stochastic optimization: Simulated annealing

In this work, we adopted simulated annealing (SA) [13, 14], one of the prime stochastic optimization techniques to decipher the correct kinetic parameter set associated with the model. The algorithm of SA was developed using the notion adopted in metallurgy. In the metallurgical annealing process, a molten mixture of metals by quickly lowering the temperature leads to a defective crystal structure of the target alloy, far from the minimum Gibbs free energy state. Starting from a high temperature, cooling must be slow when approaching the recrystallization temperature to obtain a nearly-perfect defect-free crystal, which is a crystal close to the minimum energy state.

In analogy with the metallurgical annealing, here we make use of an algorithmic temperature called annealing temperature *T*_*at*_. The extent of search space which is being sampled is determined by the magnitude of annealing temperature. Considering Boltzmann distribution to simulate the behavior of the ensemble, the probability of the energy state of the system at temperature *T* is given by *p*(*E*_0_) = exp(−*E*_0_/*k*_*B*_*T*)/*Z*(*T*), where *E*_0_ is the energy of the state, *k*_*B*_ is Boltzmann constant and *Z*(*T*) is the normalization factor. We define a cost function or objective function Δ to follow the progress of the search towards the solution. While carrying out the optimization, in each iteration, a small random move is applied to a random kinetic parameter, and the subsequent difference in Δ is estimated. If Δ ≤ 0 the new state is always accepted. On the other hand, if Δ > 0 the state can be accepted with some probability to escape from the local minima, using the principle of Metropolis test [15]. The transition probability of accepting the later type of solution (Δ > 0) is *F*(*T*_*at*_) = exp(−Δ/*T*_*at*_). In every iteration *F*(*T*_*at*_) is compared with a random number between 0 and 1. If the value of *F*(*T*_*at*_) is greater than the random number, the solution is accepted, otherwise rejected. The physical reasoning behind this criterion is that if *F*(*T*_*at*_) is greater than the invoked random number between 0 and 1, the move is more probable than a random event which in the present case is the generation of a random number. If a solution is rejected, then another neighbouring solution is generated and evaluated. The persistence of each temperature level regulates the number of iterations at a particular temperature. The temperature reduction takes place during the search process according to a cooling schedule, and the process terminates after reaching a specified (target) lowest temperature.

### Sensitivity analysis: Correlation coeffcient

Presence of variability in the experimental data often brings in the complication in the proper estimation of kinetic parameters associated with model biological systems. To identify and subsequent quantification of the relevant model parameters we adopt the method of sensitivity analysis [16–20]. In the present report, we quantify the sensitivity of each parameter of the model using the measure of a different correlation coefficient.

Here, we calculate three types of correlation coefficients, namely Pearson correlation coefficient (CC), Spearman rank correlation coefficient (RCC) and, partial rank correlation coefficient (PRCC). Usage of CC is appropriate in case of linear dependence, but in the case of nonlinear monotonic dependence, RCC gives more accurate results. In RCC, the correlation coefficient is calculated after a rank transformation of the data set. Correlation of a particular input parameter (from a set of parameters) with the output while excluding the effect of rest of the parameters is known as PRCC. PRCC between a particular input and the output thus excludes any effect of other model inputs. In other words, it is cleaned of any correlation between multiple inputs [16, 18]. Calculation of PRCC also takes care of sensitivity measure for the nonlinear but monotonic relationship between rank transformed data, hence making it the most efficient and reliable metric. It is important to mention that the strength of dependency between the input and the output is measured by the magnitude of the correlation coefficient, and it varies from -1 to +1. A low value signifies weak dependency, whereas a high value represents strong dependence. The negative values indicate anti-correlation between the input and the output. In the present report, the absolute magnitude of the correlation coefficient is a measure for the evaluation of the sensitivity of the input with respect to the output.

To quantify the correlation coefficients, all the optimized parameters reported in Table 1 have been perturbed randomly in order to solve the kinetic equations (see Eqs. (S41-S58) in S1 Text). The random perturbation is drawn from a Gaussian distribution. The mean and the variance of the Gaussian distribution is the base value of the parameter and is ±5% (and ±10%, see S2–S6 Tables) of the base value, respectively. The kinetic equations with a perturbed set of parameters are solved numerically using numerical ODE solver NDSolve of Mathematica (version 11.3, Wolfram Inc.) till the system reaches steady state. After each simulation, the steady state value of GFP is collected to figure out its dependency on a particular parameter value. To calculate the correlation coefficients between the parameter-GFP pair, we carried out 10^6^ independent simulation.

**Table 1:** List of optimized parameters associated with DevR regulated gene expression. Here, *x* ± *y* stands for the value of optimized parameter *x* with standard deviation *y*. The standard deviation is evaluated using the data of 10^3^ independent SA simulations. Parameter values tabulated in Set 1 and Set 2 are due to different sets of random initial conditions. Parameters shown under Mean is the average values of Set 1 and Set 2.

Parameter	Set 1	Set 2	Mean	Unit
*k*_*srp*_	(2.89 ± 0.54) × 10^−3^	(3.41 ± 0.68) × 10^−3^	(3.15 ± 0.61) × 10^−3^	nM s^−1^
*k*_*drp*_	(5.16 ± 1.13) × 10^−5^	(4.75 ± 0.88) × 10^−5^	(4.96 ± 1.01) × 10^−5^	s^−1^
*k*_*b*1_	(3.81 ± 0.54) × 10^−7^	(3.13 ± 0.69) × 10^−7^	(3.47 ± 0.62) × 10^−7^	nM^−1^s^−1^
*k*_*u*1_	(4.12 ± 0.91) × 10^−8^	(4.72 ± 0.88) × 10^−8^	(4.42 ± 0.90) × 10^−8^	s^−1^
*k*_*b*2_	(3.48 ± 0.45) × 10^−7^	(3.40 ± 0.75) × 10^−7^	(3.44 ± 0.60) × 10^−7^	nM^−1^s^−1^
*k*_*u*2_	(4.50 ± 0.99) × 10^−8^	(5.59 ± 1.05) × 10^−8^	(5.05 ± 1.02) × 10^−8^	s^−1^
*k*_*b*3_	(3.43 ± 0.66) × 10^−7^	(2.81 ± 0.55) × 10^−7^	(3.12 ± 0.61) × 10^−7^	nM^−1^s^−1^
*k*_*u*3_	(5.58 ± 1.17) × 10^−7^	(4.44 ± 0.67) × 10^−7^	(5.01 ± 0.92) × 10^−7^	s^−1^
*k*_*b*4_	(4.76 ± 0.84) × 10^−7^	(3.72 ± 0.67) × 10^−7^	(4.24 ± 0.76) × 10^−7^	nM^−1^s^−1^
*k*_*u*4_	(4.49 ± 0.72) × 10^−7^	(5.84 ± 0.89) × 10^−7^	(5.17 ± 0.81) × 10^−7^	s^−1^
*k*_*b*5_	(3.80 ± 0.80) × 10^−7^	(3.00 ± 0.56) × 10^−7^	(3.40 ± 0.68) × 10^−7^	nM^−1^s^−1^
*k*_*u*5_	(4.28 ± 0.76) × 10^−7^	(4.13 ± 0.71) × 10^−7^	(4.21 ± 0.74) × 10^−7^	s^−1^
*k*_*b*6_	(4.25 ± 0.72) × 10^−7^	(4.77 ± 0.80) × 10^−7^	(4.51 ± 0.76) × 10^−7^	nM^−1^s^−1^
*k*_*u*6_	(4.59 ± 0.78) × 10^−7^	(5.85 ± 0.89) × 10^−7^	(5.22 ± 0.84) × 10^−7^	s^−1^
*k*_*b*7_	(3.52 ± 0.73) × 10^−7^	(2.85 ± 0.49) × 10^−7^	(3.19 ± 0.61) × 10^−7^	nM^−1^s^−1^
*k*_*u*7_	(5.30 ± 0.90) × 10^−7^	(6.33 ± 0.97) × 10^−7^	(5.82 ± 0.94) × 10^−7^	s^−1^
*k*_*b*8_	(4.12 ± 0.71) × 10^−7^	(3.66 ± 0.55) × 10^−7^	(3.89 ± 0.63) × 10^−7^	nM^−1^s^−1^
*k*_*u*8_	(5.66 ± 1.05) × 10^−7^	(3.98 ± 0.58) × 10^−7^	(4.82 ± 0.82) × 10^−7^	s^−1^
*k*_*b*9_	(3.53 ± 0.64) × 10^−7^	(6.45 ± 1.11) × 10^−7^	(4.99 ± 0.88) × 10^−7^	nM^−1^s^−1^
*k*_*u*9_	(4.19 ± 0.74) × 10^−7^	(4.47 ± 0.69) × 10^−7^	(4.33 ± 0.72) × 10^−7^	s^−1^
*k*_*sm*1_	(0.60 ± 0.21) × 10^−3^	(1.20 ± 0.13) × 10^−3^	(0.9 ± 0.17) × 10^−3^	nM s^−1^
*k*_*sm*2_	(7.12 ± 1.54) × 10^−4^	(4.10 ± 0.86) × 10^−4^	(5.61 ± 1.20) × 10^−4^	nM s^−1^
*k*_*sm*3_	(6.19 ± 0.28) × 10^−3^	(5.57 ± 1.56) × 10^−3^	(5.88 ± 0.92) × 10^−3^	nM s^−1^
*k*_*sm*4_	(4.39 ± 0.85) × 10^−3^	(4.83 ± 0.71) × 10^−3^	(4.61 ± 0.78) × 10^−3^	nM s^−1^
*k*_*sm*5_	(0.46 ± 0.09) × 10^−3^	(1.21 ± 0.36) × 10^−3^	(0.84 ± 0.23) × 10^−3^	nM s^−1^
*k*_*sm*6_	(1.14 ± 0.39) × 10^−3^	(0.92 ± 0.34) × 10^−3^	(1.03 ± 0.37) × 10^−3^	nM s^−1^
*k*_*sm*7_	(7.14 ± 1.00) × 10^−3^	(5.13 ± 0.68) × 10^−3^	(6.14 ± 0.84) × 10^−3^	nM s^−1^
*k*_*sm*8_	(6.13 ± 1.08) × 10^−4^	(3.97 ± 0.60) × 10^−4^	(5.05 ± 0.84) × 10^−4^	nM s^−1^
*k*_*sm*9_	(4.44 ± 0.73) × 10^−4^	(4.42 ± 0.71) × 10^−4^	(4.43 ± 0.72) × 10^−4^	nM s^−1^
*k*_*sm*10_	(4.82 ± 0.96) × 10^−5^	(3.88 ± 0.53) × 10^−5^	(4.35 ± 0.75) × 10^−5^	nM s^−1^
*k*_*sm*11_	(3.79 ± 0.76) × 10^−3^	(3.13 ± 0.54) × 10^−3^	(3.46 ± 0.65) × 10^−3^	nM s^−1^
*k*_*sm*12_	(6.00 ± 1.08) × 10^−5^	(5.87 ± 0.93) × 10^−5^	(5.94 ± 1.01) × 10^−5^	nM s^−1^
*k*_*sm*13_	(4.82 ± 0.86) × 10^−5^	(6.06 ± 0.90) × 10^−5^	(5.44 ± 0.88) × 10^−5^	nM s^−1^
*k*_*sm*14_	(5.97 ± 1.17) × 10^−5^	(6.00 ± 0.90) × 10^−5^	(5.99 ± 1.04) × 10^−5^	nM s^−1^
*k*_*sm*15_	(4.95 ± 0.83) × 10^−4^	(4.72 ± 0.73) × 10^−4^	(4.84 ± 0.78) × 10^−4^	nM s^−1^
*k*_*sm*16_	(5.79 ± 1.05) × 10^−4^	(4.12 ± 0.73) × 10^−4^	(4.96 ± 0.89) × 10^−4^	nM s^−1^
*k*_*sm*17_	(6.26 ± 1.17) × 10^−3^	(5.94 ± 0.96) × 10^−3^	(6.10 ± 0.97) × 10^−3^	nM s^−1^
*k*_*sm*18_	(5.24 ± 0.91) × 10^−4^	(7.26 ± 1.11) × 10^−4^	(6.25 ± 1.01) × 10^−4^	nM s^−1^
*k*_*sm*19_	(4.38 ± 0.83) × 10^−3^	(4.93 ± 0.83) × 10^−3^	(4.66 ± 0.83) × 10^−3^	nM s^−1^
*k*_*dm*_	(4.34 ± 0.86) × 10^−4^	(3.74 ± 0.60) × 10^−4^	(4.04 ± 0.73) × 10^−4^	s^−1^
*k*_*sg*_	(5.03 ± 0.92) × 10^−4^	(3.79 ± 0.55) × 10^−4^	(4.41 ± 0.74) × 10^−4^	nM s^−1^
*k*_*dg*_	(2.75 ± 0.57) × 10^−5^	(2.62 ± 0.51) × 10^−5^	(2.69 ± 0.54) × 10^−5^	s^−1^

### Sensitivity amplification

To further check the role of kinetic parameters in gene expression, we employ the measure of sensitivity amplification [21–23]. Sensitivity amplification is the relative percentage change in response with respect to the percentage change in stimulus. In the present study, a change in the GFP level is considered as a response while a change in the kinetic rate parameters is considered as a variation of the stimulus. Considering this we define sensitivity amplification

$$A_{S}=\frac{\Delta GFP/GFP^{i}}{\Delta k/k^{i}},$$

with Δ*GFP* = *GFP*^*f*^ − *GFP*^*i*^ and Δ*k* = *k*^*f*^ − *k*^*i*^ where *i* and *f* stand for the initial and final value, respectively.

## Results and discussions

### Optimization of the kinetic parameters

One of the main goals of the current work is to obtain the right set of kinetic parameters relevant to DevR controlled gene expression. In this context, it is essential to mention that parameters related to the current project are unavailable in the literature. To the best of our knowledge, only experimental information we have at our disposal is gene expression of wild type and some mutants [7, 24]. Keeping this in mind, we carried out stochastic optimization of the full kinetic parameter set. During simulation optimization of each parameter *k* (say) is done using the relation

$$k^{\prime }=k+k\times {\left(-1\right)}^{n}\times \delta \times r_{n},$$

with *k*′ being the updated value of *k*. Here, *n* is a random integer, *δ* is the amplitude of allowed change of a selected parameter which in our case lies between 0.01 to 0.05, and *r*_*n*_ is a random integer between 0 and 1. As mentioned in Sec 2.2 we calculated a cost function or objective function for the new set of updated variables in each iteration. For *Rv3134c* and *hspX* we use the cost function

$$\Delta =\sum_{i=1}^{N}{\left(G_{i}^{\text{exp}}-G_{i}^{T_{at}}\right)}^{2}.$$

Here $G_{i}^{\text{exp}}$ is the experimental (target) GFP value and $G_{i}^{T_{at}}$ is the simulated GFP value at the annealing temperature *T*_*at*_ evaluated at the *i*-th step of the simulation. Due to common sharing of the promoter we use the following cost function for *narK2-Rv1738* system

$$\Delta =\alpha \sum_{i=1}^{N}{\left(G_{i}^{\text{exp}}-G_{i}^{T_{at}}\right)}^{2}+\beta \sum_{i=1}^{N}{\left(G_{i}^{\text{exp}}-G_{i}^{T_{at}}\right)}^{2},$$

where *α* and *β* are scalar weights. In the present problem, we set *α* = *β* = 1.

**Fig 2:**
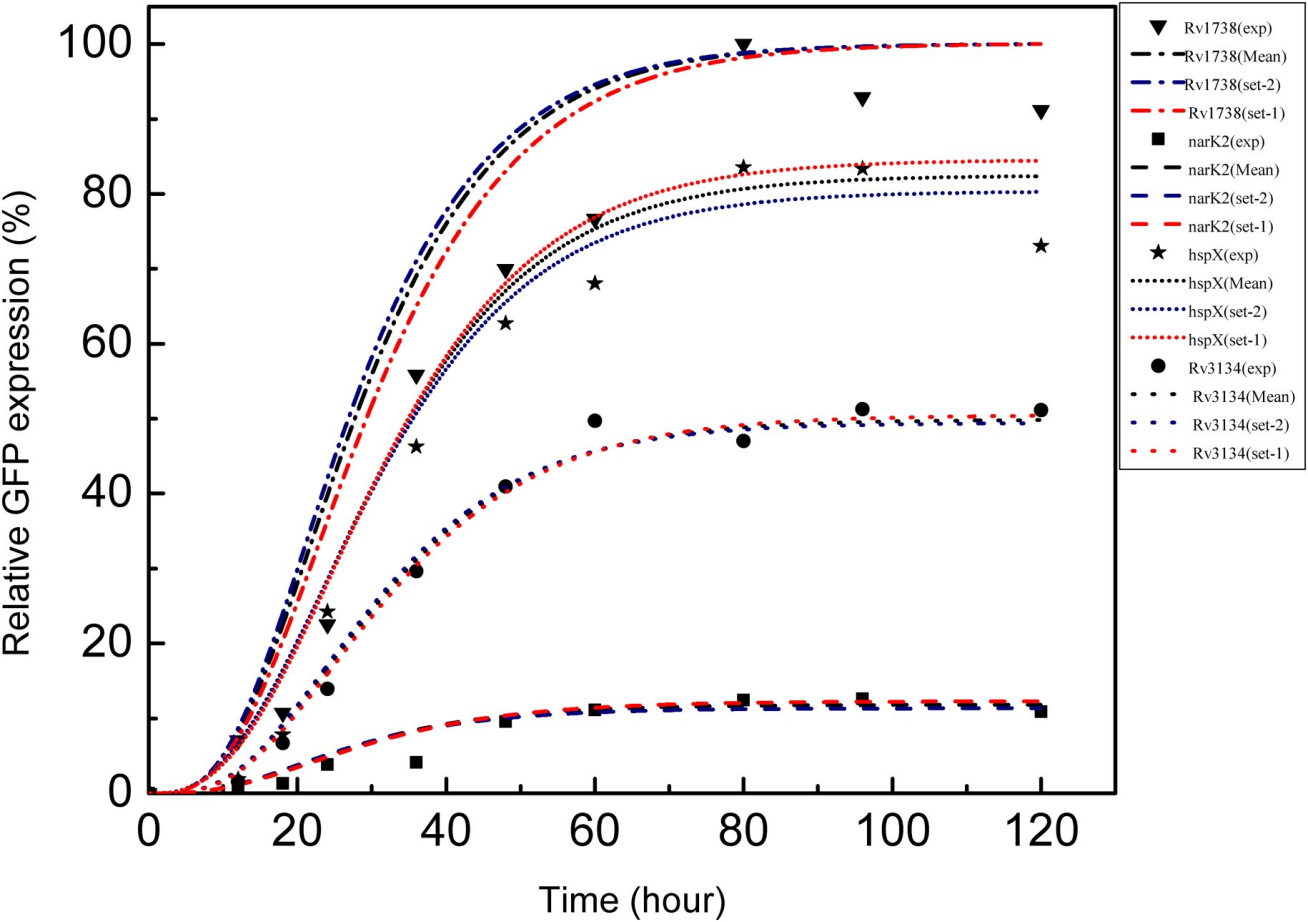
Temporal gene expression. Temporal GFP expression of *Rv3134c*, *hspx*, *narK2* and *Rv1738*. The symbols are taken from Chauhan and Tyagi [7] and the lines are generated using optimized set of parameters shown in Table 1. Red and blue lines are drawn using Set 1 and Set 2, respectively. The black lines are due to Mean values reported in Table 1.

**Fig 3:**
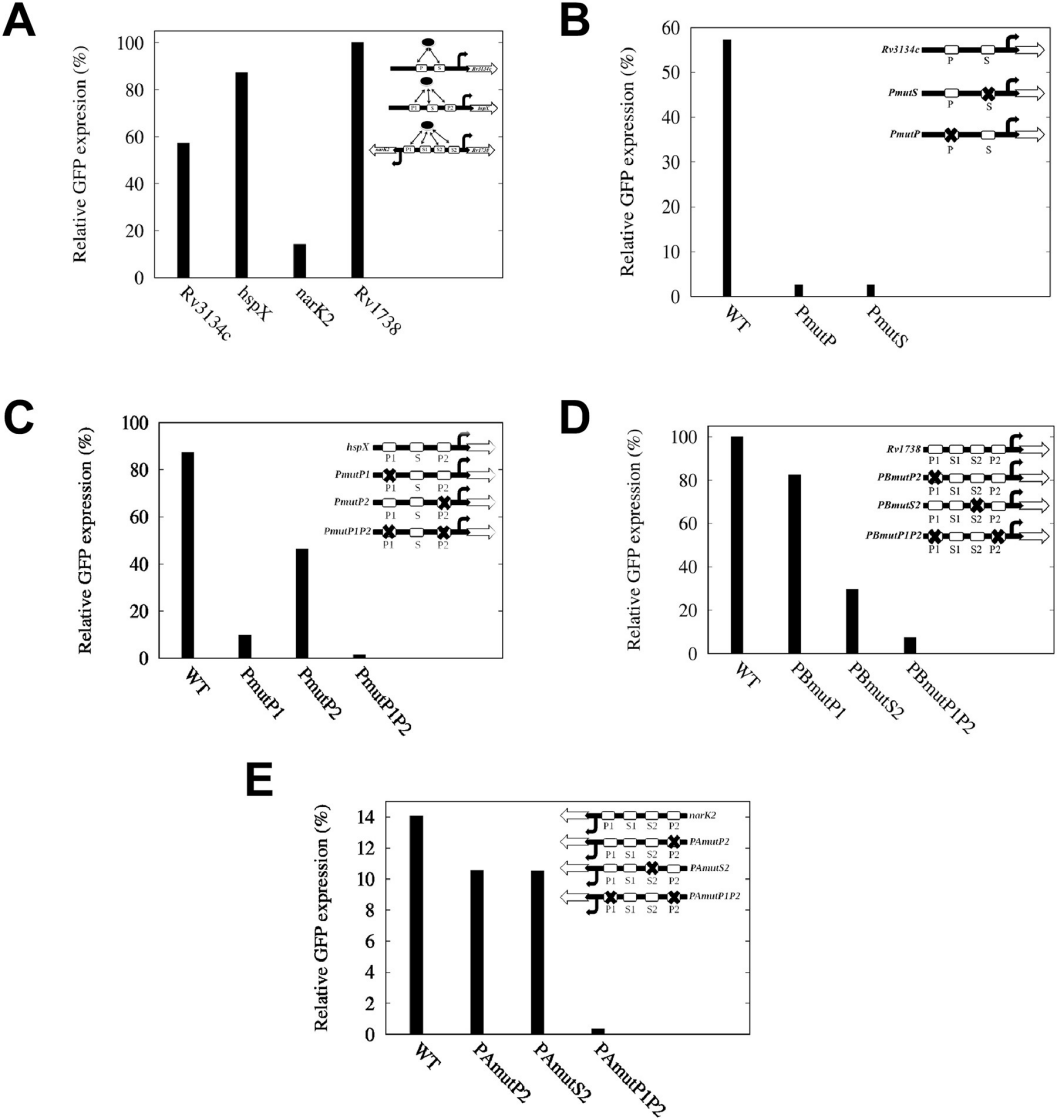
Relative gene expression at steady state. **A**.*Rv3134c*, *hspX*, *narK2* and *Rv1738*. **B**. *Rv3134c* and mutants. **C**. *hspX* and mutants. **D**. *Rv1738* and mutants, and **E**. *narK2* and mutants.

**Fig 4:**
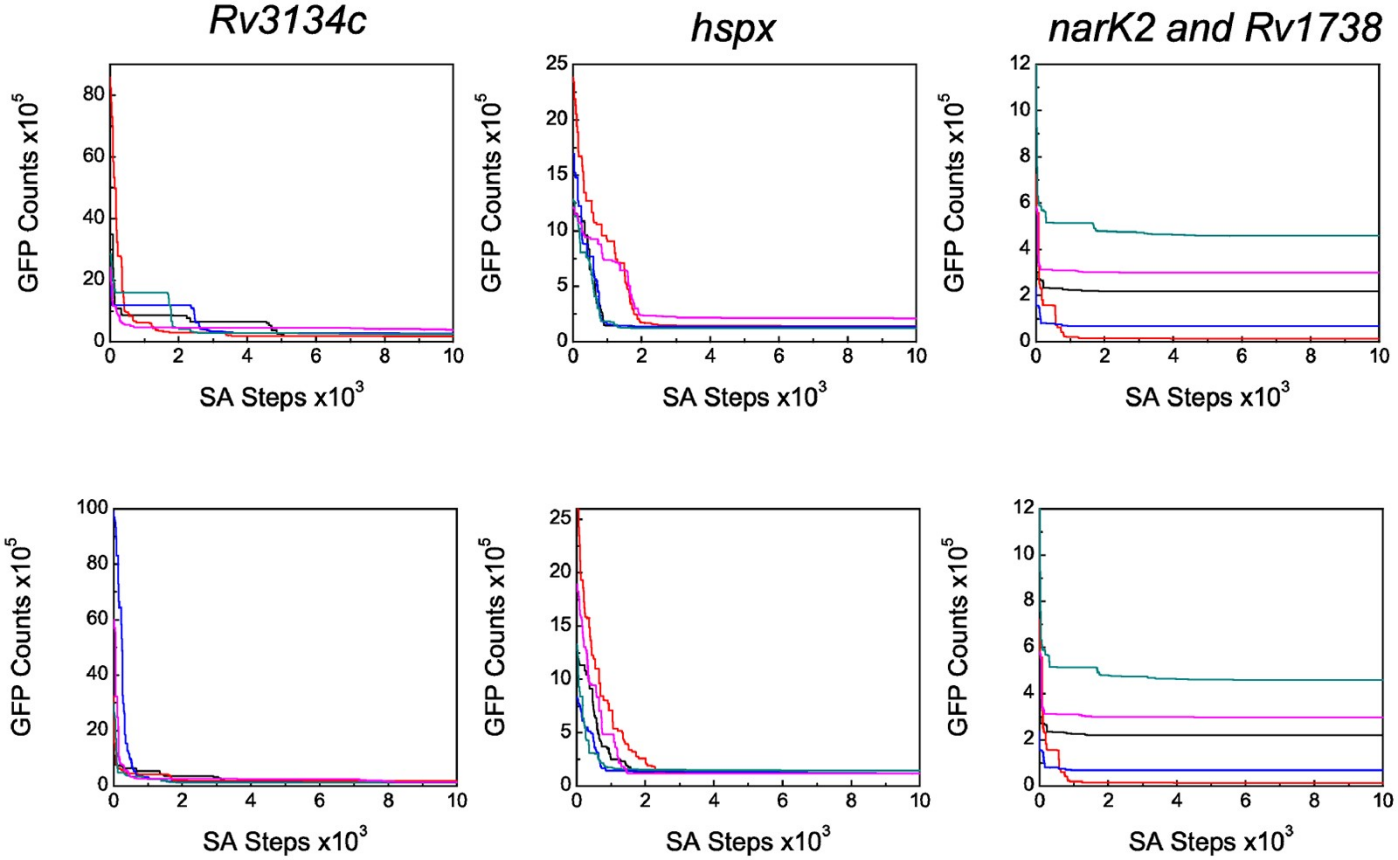
Evolution of cost function. The cost function of GFP counts as a function of SA steps associated with *Rv3134c*, *hspx*, *narK2* and *Rv1738*. The five solid lines are generated from five different SA runs and are representatives of 10^3^ simulation. Top and bottom panels are due to Set 1 and Set 2 parameters, respectively.

Following the above structures of the cost function, we carried out the simulation. Initially, we ascribed random initial values to the model parameters to execute the simulation. To check whether different sets of initial conditions lead to a unique parameter set, we performed simulation using two different random sets of initial conditions (please see the end of S1 Text). The two resultant optimized parameter sets are given in Table 1. In addition, Table 1 also shows the mean of the two parameter sets. The optimized parameters were then used to generate the experimental GFP expression profiles of all the target genes (see Fig 2) reported by Chauhan and Tyagi [7]. The corresponding steady state levels of GFP are shown in Fig 3A. We note that the parameter values reported in these two sets although look different, they, however, generate similar kind of temporal profiles as shown in Fig 2. The profiles of cost function and evolution of kinetic rate parameters are shown in Fig 4 and S1–S3 Figs, respectively. We note that the experimental values plotted in Fig 2 show reduced expression at later time points (∼ 90−120 hrs). Multiple reasons may cause such reduction, e.g., cell death due to limited resources, dilution effect, etc. [25].

To generate the desired optimized set of parameters, we carried out 10^3^ independent SA runs. In each independent simulation, the initial annealing temperature *T*_*at*_ was set at 300 along with 1% rate of cooling (annealing schedule). The number of SA steps in each simulation was 10^4^. Multiple SA runs are essential because the upgradation of parameter values in the model is done by using random numbers and in a given run, there is a finite possibility that the updated parameters are not able to cause a reduction in the cost function value by a substantial amount. However, if a large number of SA runs are executed as has been done in the present study, precise upgradation will happen in certain trajectories with the associated decrease in cost function quickly and towards the desired solution.

### Sensitivity of the kinetic parameters

After optimization of the full kinetic parameter set, we focus on finding out the sensitivity of the same in connection to gene expression. To this end, we first quantify the correlation between the GFP level of the four target genes with the synthesis (*k*_*srp*_) and degradation (*k*_*drp*_) rate of the transcription factor *R*_*P*_. We also calculate the correlation of the GFP level with the synthesis (*k*_*sg*_) and degradation (*k*_*dg*_) rate of the GFP itself. As discussed in Sec 2.3, all the parameters have been perturbed randomly to solve the kinetic equations (using the perturbed set of parameters). A Representative of 10^3^ simulation data are shown as scatter plots in S4 Fig. The nature of scatter plots suggests that the synthesis and the degradation rate of the transcription factor have a weak effect on the GFP level. However, the synthesis and degradation rate of GFP shows a prominent positive and negative correlation, respectively. To quantify the correlation, we calculate CC, RCC and PRCC for all the four genes and are tabulated in Table 2. Here the correlation values have been calculated using 10^6^ independent trajectories. While quantifying the correlation values, we have used both the parameter sets (i.e., Set 1 and Set 2) shown in Table 1. As a result, each measure of correlation coefficients has three entries in Table 2. We adopted the same strategy for measuring correlation values for other model parameters. The correlation values listed in Table 2 suggests an ordering of the target genes output (GFP level) for each of the four parameters (*k*_*srp*_, *k*_*drp*_, *k*_*sg*_ and *k*_*dg*_)

$$\begin{array}{c} k_{srp}:Rv3134c<narK2∼Rv1738∼hspX,\\ k_{drp}:Rv3134c<narK2∼Rv1738∼hspX,\\ k_{sg}:Rv3134c∼narK2∼Rv1738∼hspX,\\ k_{dg}:Rv3134c∼narK2∼Rv1738∼hspX. \end{array}$$

Here we note that the synthesis rate *k*_*sg*_ is the translation rate of GFP from its mRNA. In the present model, *k*_*sg*_ is the same for all the target genes, which gets reflected in the almost equal correlation between *k*_*sg*_ and GFP. On a similar note correlation between *k*_*dg*_ and GFP becomes equal. The PRCC values reported in Table 2 are higher than CC and RCC values as calculation of PRCC excludes the effect of other parameters in the calculation.

**Table 2:** CC, RCC, PRCC values for synthesis and degradation rate of four genes using 5% perturbation.

		*Rv3134c*			*hspX*			*narK2*			*Rv1738*		
	Parameter	CC	RCC	PRCC	CC	RCC	PRCC	CC	RCC	PRCC	CC	RCC	PRCC
Set 1	*k*_*srp*_	0.010	0.010	0.028	0.029	0.028	0.098	0.025	0.023	0.083	0.027	0.026	0.100
	*k*_*drp*_	-0.006	-0.004	-0.023	-0.020	-0.021	-0.100	-0.026	-0.025	-0.082	-0.026	-0.025	-0.088
	*k*_*sg*_	0.516	0.500	0.886	0.536	0.523	0.895	0.549	0.534	0.900	0.546	0.531	0.898
	*k*_*dg*_	-0.520	-0.504	-0.885	-0.539	-0.521	-0.895	-0.551	-0.534	-0.900	-0.547	-0.530	-0.898
Set 2	*k*_*srp*_	-0.001	-0.001	0.014	0.022	0.021	0.084	0.016	0.016	0.068	0.019	0.019	0.074
	*k*_*drp*_	0.002	0.003	-0.018	-0.021	-0.020	-0.077	-0.018	-0.017	-0.068	-0.017	-0.017	-0.078
	*k*_*sg*_	0.523	0.508	0.888	0.543	0.529	0.897	0.551	0.536	0.900	0.547	0.532	0.899
	*k*_*dg*_	-0.525	-0.507	-0.888	-0.546	-0.527	-0.896	-0.549	-0.531	-0.899	-0.545	-0.527	-0.897
Mean	*k*_*srp*_	0.005	0.005	0.021	0.026	0.025	0.091	0.021	0.020	0.076	0.023	0.023	0.087
	*k*_*drp*_	-0.002	-0.001	-0.021	-0.021	-0.021	-0.089	-0.022	-0.021	-0.075	-0.022	-0.021	-0.083
	*k*_*sg*_	0.520	0.504	0.887	0.540	0.526	0.896	0.550	0.535	0.900	0.574	0.532	0.899
	*k*_*dg*_	-0.523	-0.506	-0.887	-0.543	-0.524	-0.896	-0.550	-0.533	-0.900	-0.546	-0.529	-0.898

Having figured out the sensitivity of the parameters associated with the transcription factor *R*_*P*_, we focus on the parameters associated with the four target genes. The degradation rate of GFP (*k*_*dg*_) shows the maximum level of correlation (negative) with GFP itself compared to the other parameters. We thus exclude *k*_*dg*_ while looking at the sensitivity of other parameters related to gene expression.

#### Two binding sites act co-oepratively in *RV3134c* expression

First, we analyse the sensitivity of the parameters related to *Rv3134c*. Using the previous strategy, we quantified the correlation between the parameters and the GFP level (Table 3). Here, the relative ordering of the parameters in terms of correlation values (absolute) are

$$k_{sm3}\gg k_{sm1}∼k_{sm2}>k_{b1}∼k_{b2}\gg k_{u1}∼k_{u2}.$$

The above ordering suggests that both the primary (P) and secondary (S) binding sites are necessary for full activation of the *Rv3134c* gene. Such necessity gets reflected in the high correlation value associated with the parameter *k*_*sm*3_ taking care of co-operativity driven mRNA production. This is in agreement with the maximum level of GFP expression in the wild type strain reported by Chauhan and Tyagi [26]. Furthermore, the ordering *k*_*sm*3_ ≫ *k*_*sm*1_ ∼ *k*_*sm*2_ suggests that mutation of one of the binding sites (P or S) reduces the correlation of *k*_*sm*1_ and *k*_*sm*2_ with the output (GFP). During the experiment when either of the binding sites is mutated, the mutants pmutP and pmutS show ∼25 fold less GFP expression compared to the wild type strain (Fig 3B) [26]. Correlation associated with the binding parameters *k*_*b*1_ and *k*_*b*2_ comes next and have minimal effect on the GFP level. The unbinding parameters *k*_*u*1_ and *k*_*u*2_ have the least contribution with an order of magnitude lower value of (negative) correlation.

**Table 3:** CC, RCC, PRCC values for all the parameter of *Rv3134c*. The input parameters are calculated with output GFP using 5% perturbation.

	CC			RCC			PRCC		
Parameter	Set1	Set2	Mean	Set1	Set2	Mean	Set1	Set2	Mean
*k*_*dm*_	-0.521	-0.523	-0.522	-0.505	-0.506	-0.506	-0.887	-0.887	-0.887
*k*_*sm*3_	0.428	0.403	0.416	0.413	0.391	0.402	0.844	0.830	0.837
*k*_*sm*1_	0.044	0.090	0.067	0.043	0.087	0.065	0.150	0.304	0.227
*k*_*sm*2_	0.050	0.028	0.039	0.049	0.030	0.040	0.180	0.115	0.148
*k*_*b*1_	0.008	0.009	0.008	0.007	0.009	0.008	0.023	0.024	0.023
*k*_*b*2_	0.009	0.008	0.008	0.008	0.010	0.009	0.024	0.025	0.024
*k*_*u*1_	0.003	-0.007	-0.002	0.001	-0.008	-0.004	-0.000	-0.013	-0.007
*k*_*u*2_	-0.000	0.006	0.006	-0.006	-0.005	-0.005	-0.001	-0.003	-0.002

#### Binding of *R*_*P*_ to distal site P1 acts as a bottleneck for *hspX* expression

Next, we check sensitivity of the parameters associated with *hspX*. The ordering of the parameters related to expression of *hspX* in terms of correlation values (absolute) (see Table 4) are

$$k_{sm4}>k_{sm7}>k_{sm5}>k_{sm6}\gg k_{b3}∼k_{u3}>k_{b5}∼k_{u4}∼k_{u5}∼k_{b4}.$$

In *hspX*, the synthesis rate *k*_*sm*4_ from the activated distal primary binding site P1 shows a maximum correlation with the output (GFP) compared to the other synthesis rates associated with *hspX*. On the other hand, the activation and inactivation rates (*k*_*b*3_ and *k*_*u*3_) related to the formation of active P1 (P1*) show a lesser correlation. These two opposing factors work together to determine the effective contribution of distal binding site P1 in the expression of *hspX* as reported by Chauhan et al. [6]. In other words, *K*_*D*_ (= *k*_*u*_/*k*_*b*_) value associated with the distal binding site P1 acts as a bottleneck for its proper functionality. Due to this reason, the mutant pmutP1 (mutation at P1) showed ∼ 30% of the wild type GFP expression, as reported by Park et al. [24] (Fig 3C).

**Table 4:** CC, RCC, PRCC values for all the parameter of *hspX*. The input parameters are calculated with output GFP using 5% perturbation.

	CC			RCC			PRCC		
Parameter	Set1	Set2	Mean	Set1	Set2	Mean	Set1	Set2	Mean
*k*_*dm*_	-0.538	-0.547	-0.543	-0.520	-0.530	-0.525	-0.895	-0.897	-0.896
*k*_*sm*4_	0.286	0.227	0.257	0.276	0.219	0.248	0.729	0.639	0.684
*k*_*sm*7_	0.182	0.218	0.200	0.175	0.211	0.193	0.562	0.630	0.596
*k*_*sm*5_	0.048	0.043	0.046	0.046	0.041	0.044	0.177	0.154	0.166
*k*_*sm*6_	0.014	0.057	0.036	0.015	0.055	0.035	0.078	0.205	0.142
*k*_*b*3_	0.015	0.012	0.014	0.013	0.013	0.013	0.053	0.049	0.051
*k*_*u*3_	-0.014	-0.007	-0.011	-0.013	-0.007	-0.010	-0.052	-0.030	-0.041
*k*_*b*5_	0.010	0.011	0.011	0.011	0.010	0.011	0.032	0.014	0.023
*k*_*u*4_	-0.001	-0.005	-0.003	-0.000	-0.004	-0.002	-0.022	-0.021	-0.022
*k*_*u*5_	-0.002	-0.009	-0.006	-0.003	-0.009	-0.006	-0.023	-0.021	-0.022
*k*_*b*4_	0.007	0.002	0.005	0.004	0.003	0.004	0.019	0.020	0.020

The next parameter that shows high correlation with GFP is *k*_*sm*7_ which takes care of synthesis of GFP due to the cooperative effect of all the three binding sites (P1, S and P2) of *hspX*. The synthesis rates *k*_*sm*5_ and *k*_*sm*6_ due to P2 and S, respectively, come next. As P2 lies close to the transcription start point (TSP) of *hspX*, the GFP synthesis rate due to P2 is higher than that of S. It is important to note that, mutation at P2 (pmutP2) shows ∼ 53% of the wild type expression. Thus mutation at both primary binding sites P1 and P2 (pmutP1P2) shows only ∼ 12% of wild type expression and agrees with Park et al. [24]. The correlation values due to binding (*k*_*b*4_ & *k*_*b*5_) and unbinding (*k*_*u*4_ & *k*_*u*5_) rates with GFP for the activation of P2 and S show that preference of *R*_*P*_ is almost equal for P2 and S due to their close proximity.

#### Promoter sharing reverses the role of binding sites in *narK2-Rv1738* system

Finally, we analyse the sensitivity of the parameters related to *narK2-Rv1738* system. For these pair of genes we quantify the correlation coefficients (Tables 5 and 6). The ordering of the parameters related to *narK2* in terms of absolute correlation values are (see Table 5)

$$\begin{array}{c} k_{sm8}>k_{sm16}>k_{sm18}\gg k_{sm14}∼k_{sm12}>k_{sm10}>k_{b6}∼k_{b7}∼\\ k_{u6}∼k_{u7}>k_{b9}∼k_{u8}∼k_{b8}∼k_{u9}. \end{array}$$

In *narK2*, the parameter with the highest correlation coefficient is *k*_*sm*8_, related to transcription from the primary site P1. Thus deletion of P1 results in almost zero expression in the mutant pAmutP1 [7]. The next sensitive parameter is *k*_*sm*16_ that takes care of transcription from both P1 and S1. The parameter *k*_*sm*18_ is related to the transcription from the secondary binding sites P2 and S2. The low value of *k*_*sm*18_ compared to *k*_*sm*8_ and *k*_*sm*16_ is due to the distal nature of P2 and S2 from the TSP of *narK2*. The correlation ordering of *k*_*sm*8_ together with *k*_*sm*16_ and *k*_*sm*18_ shows that contribution of P2 and S2 in the expression of *narK2* is minimal and both are unable to rescue the low expression profile of pAmutP1. Due to the distal nature of P2 and S2 single mutation results in almost similar kind of expression in pAmutP2 and pAmutS2, respectively [7] (see Fig 3E).

**Table 5:** CC, RCC, PRCC values for all the parameter of *narK2*. The input parameters are calculated with output GFP using 5% perturbation.

	CC			RCC			PRCC		
Parameter	Set1	Set2	Mean	Set1	Set2	Mean	Set1	Set2	Mean
*k*_*dm*_	-0.552	-0.546	-0.549	-0.535	-0.530	-0.533	-0.900	-0.900	-0.900
*k*_*sm*8_	0.154	0.235	0.195	0.147	0.226	0.187	0.487	0.655	0.571
*k*_*sm*16_	0.182	0.128	0.155	0.175	0.123	0.149	0.558	0.436	0.497
*k*_*sm*18_	0.171	0.135	0.153	0.165	0.130	0.148	0.528	0.445	0.487
*k*_*sm*14_	0.016	0.021	0.019	0.015	0.020	0.018	0.067	0.075	0.071
*k*_*sm*12_	0.015	0.018	0.017	0.015	0.015	0.015	0.065	0.071	0.068
*k*_*sm*10_	0.010	0.011	0.012	0.010	0.010	0.010	0.053	0.041	0.047
*k*_*b*6_	0.006	0.008	0.007	0.006	0.009	0.008	0.029	0.025	0.027
*k*_*b*7_	0.008	0.009	0.009	0.007	0.008	0.008	0.019	0.032	0.026
*k*_*u*6_	-0.006	-0.005	-0.006	-0.005	-0.006	-0.006	-0.024	-0.016	-0.020
*k*_*u*7_	-0.002	-0.009	-0.006	-0.001	-0.009	-0.005	-0.008	-0.031	-0.020
*k*_*b*9_	0.007	0.000	0.004	0.006	0.002	0.004	0.022	0.013	0.018
*k*_*u*8_	-0.007	-0.000	-0.004	-0.006	-0.001	-0.004	-0.020	-0.009	-0.015
*k*_*b*8_	0.010	0.007	0.009	0.009	0.006	0.008	0.014	0.012	0.013
*k*_*u*9_	0.001	0.001	0.001	0.000	-0.000	0.000	-0.013	-0.005	-0.009

**Table 6:** CC, RCC, PRCC values for all the parameter of *Rv1738*. The input parameters are calculated with output GFP using 5% perturbation.

	CC			RCC			PRCC		
Parameter	Set1	Set2	Mean	Set1	Set2	Mean	Set1	Set2	Mean
*k*_*dm*_	-0.548	-0.550	-0.549	-0.529	-0.531	-0.530	-0.899	-0.898	-0.899
*k*_*sm*19_	0.219	0.221	0.220	0.208	0.213	0.211	0.632	0.625	0.629
*k*_*sm*17_	0.153	0.177	0.165	0.148	0.171	0.160	0.494	0.552	0.523
*k*_*sm*11_	0.139	0.113	0.126	0.135	0.108	0.122	0.453	0.384	0.419
*k*_*sm*15_	0.016	0.017	0.017	0.015	0.017	0.016	0.070	0.065	0.068
*k*_*sm*9_	0.020	0.021	0.021	0.020	0.020	0.020	0.060	0.062	0.061
*k*_*b*7_	0.006	0.016	0.011	0.004	0.015	0.010	0.035	0.039	0.037
*k*_*u*7_	-0.010	-0.005	-0.008	-0.009	-0.004	-0.006	-0.026	-0.033	-0.030
*k*_*u*8_	-0.005	-0.003	-0.004	-0.006	-0.003	-0.005	-0.019	-0.015	-0.017
*k*_*b*6_	0.003	0.004	0.004	0.003	0.004	0.004	0.016	0.011	0.014
*k*_*u*6_	-0.000	-0.007	-0.004	-0.001	-0.006	-0.004	-0.016	-0.011	-0.014
*k*_*b*9_	0.004	0.001	0.003	0.003	0.001	0.002	0.018	0.009	0.014
*k*_*b*8_	0.003	0.003	0.003	0.003	0.002	0.003	0.017	0.008	0.013
*k*_*u*9_	-0.005	-0.003	-0.004	-0.006	-0.002	-0.004	-0.017	-0.005	-0.011
*k*_*sm*13_	0.005	0.001	0.003	0.005	0.000	0.003	0.007	0.009	0.008

Both the parameters *k*_*b*8_ and *k*_*u*8_ are related to the activation of the secondary binding site S1. Here, *k*_*b*8_ and *k*_*u*8_ together with *k*_*sm*12_ effectively controls transcription from active state of S1. Due to the close proximity of S1 and S2, transcription rate from S2 shows similar sensitivity as from S1, i.e., *k*_*sm*12_ ∼ *k*_*sm*14_. The transcription rate *k*_*sm*10_ from the distal binding site P2 shows a low correlation with the output compared to transcription from other binding sites. The parameter set *k*_*b*6_ and *k*_*u*6_ related to the activation of the primary binding site P1 shows low correlation. However, when activated the same site (P1*) starts producing the transcripts with maximum efficiency. Thus, activation of P1 serves as a bottleneck for *narK2* transcripts. On a similar note, the parameter sets (*k*_*b*7_, *k*_*u*7_) and (*k*_*b*9_, *k*_*u*9_) controls the activation of the sites P2 and S2, respectively.

In *Rv1738*, the ordering of the parameters in terms of correlation values (absolute) are (see Table 6)

$$\begin{array}{c} k_{sm19}>k_{sm17}>k_{sm11}\gg k_{sm15}∼k_{sm9}>k_{b7}∼k_{u7}\\ >k_{u8}∼k_{b6}∼k_{u6}∼k_{b9}∼k_{b8}∼k_{u9}∼k_{sm13}. \end{array}$$

The above ordering of correlation values associated with *Rv1738* suggests that the most sensitive parameter is *k*_*sm*19_, the transcription rate controlled by both P2 and S2, proximal to *Rv1738*. The parameter *k*_*sm*17_ reflects the co-operative effect of P1 and S1, however, distal binding sites for the same gene. Thus mutation at P2 drastically lowers down the GFP expression as observed in pBmutP2. Experimentally such signature is observed in the mutant pBmutP1P2 [7] (see Fig 3D). It is important to note that the said mutant also affects the GFP expression profile of *narK2*, where it has been labelled as pAmutP1P2. Both the mutants pAmutP1P2 and pBmutP1P2 are same as, as in both cases P1 and P2 site is mutated [7]. For the sake of labelling, they have been named differently. The next sensitive parameter is *k*_*sm*11_ and is due to the binding site P2 alone. Role of the binding site S2 gets reflected through *k*_*sm*15_, which appears next in the ordering. The sensitivity of transcription rate *k*_*sm*9_ from distal binding site P1 (for *Rv1738*) reduces further and has a weak role in the overall production of transcripts. The weak contribution of P1 is observed in the expression profile of the mutant pBmutP1 [7]. We again note here that the mutant pBmutP1 is same as pAmutP1 (labelled for *narK2*). Transcription rate *k*_*sm*13_ from distal secondary site S2 is least and appears at the end of ordering. In between *k*_*sm*9_ and *k*_*sm*13_, the sensitivity of different binding rates appear.

### High correlation results into high sensitivity amplification

**Fig 5:**
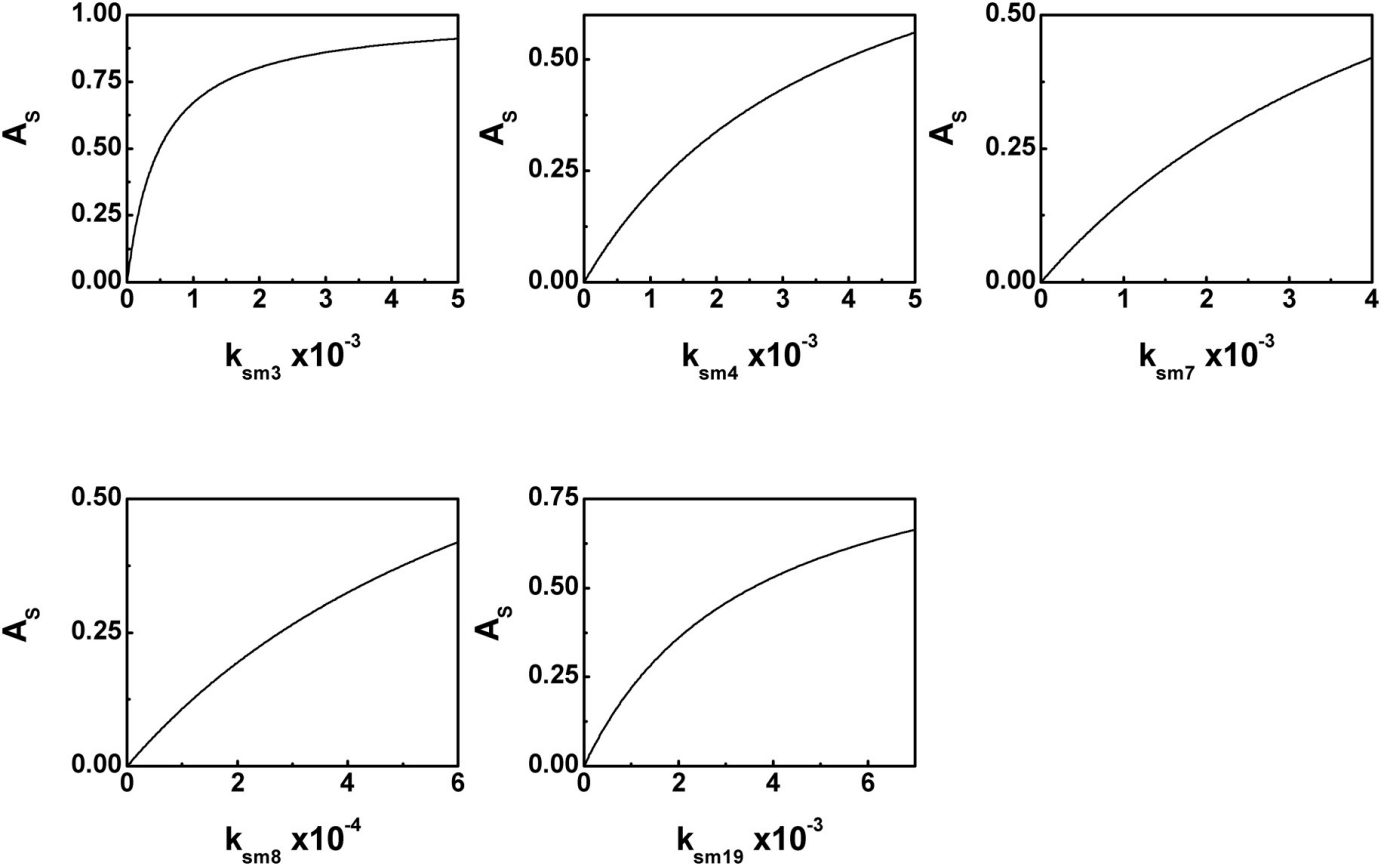
Sensitivity amplification of transcription rate. Sensitivity amplification as a function of parameters with high value of correlation coefficients. *k*_*sm*3_ is the transcription rate of *Rv3134c*. *k*_*sm*4_ and *k*_*sm*7_ are the transcription rates associated with *hspX*. *k*_*sm*8_ and *k*_*sm*19_ control the transcription of *narK2* and *Rv1738*, respectively.

The results reported in the previous subsection suggest that for each target gene, few rate parameters show a high correlation with the GFP level compared to the rest of the parameters. It is thus interesting to inspect whether the same parameter plays a crucial role in sensitivity amplification. To this end we calculate sensitivity amplification for all the parameters related to *Rv3134c*, *hspX* and *narK2-Rv1738*. The parameters with a high correlation coefficient with the output (GFP) indeed show a high level of sensitivity amplification (see Fig 5). On the other hand, the other parameters play little role in doing so (see S5–S8 Figs).

The parameter *k*_*sm*3_ represents co-operativity driven mRNA production rate in *Rv3134c* with highest value of PRCC (see Table 3). It shows highest level of sensitivity amplification compared to other parameters related to *Rv3134c*. In *hspX*, *k*_*sm*4_ and *k*_*sm*7_ show maximum correlation with output. *k*_*sm*4_ is related to gene expression regulated by P1 whereas co-operative effect of P1, S and P2 gets reflected through *k*_*sm*7_. Both these parameters show high level of sensitivity amplification of the output.

In *narK2*, the transcription rate *k*_*sm*8_ shows the maximum correlation with output and plays a decisive role in gene expression. The similar feature is observed in the calculation of sensitivity amplification. Interestingly, for *Rv1738* the proximal binding sites, P2 and S2 mainly control its expression and is reflected through a high level of transcription rate *k*_*sm*19_. A high correlation value of *k*_*sm*19_ with the output also gets reflected in sensitivity amplification.

## Conclusion

In the present work, we focused on the sensitivity of the kinetic parameters relevant to the DevR regulated gene expression in *M. tuberculosis*. To this end, a mathematical model is presented based on kinetic interactions between DevR and four target genes. We carried out stochastic optimization to obtain the physiologically relevant parameter set that can reproduce the experimental profiles. A systematic study based on sensitivity analysis is performed, which reveals the ordering of the parameters based on their correlation values with the output, i.e., GFP concentration. Together with the experimental information, our analysis provides information about the prime steps of DevR controlled gene regulation under dormancy. Sensitivity analysis reveals that the transcription rates are the crucial step in the kinetics and is further supported by sensitivity amplification. The ordering of parameters provides a guideline to tackle virulence by a proper mutation that controls the process of transcription during dormancy related gene expression in *M. tuberculosis*. The present study may work as a reference for the experimentalist to carry out further research on DevR regulated networks that are yet to be explored.

## Supporting information

S1 Text Supplementary information. Kinetic scheme and equations for phosphorylated DevR regulated gene expression. The text contains detailed scheme and differential equations for the model.
(PDF)

S1 Fig Optimization profiles of kinetic parameters associated with *Rv3134c*. The perturbed parameter is ploted as a function of SA steps. In each panel, the five colored lines originated from different SA simulation. The horizontal dotted lines is for the base parameter value reported in Table 1.
(PDF)

S2 Fig Optimization profiles of kinetic parameters associated with *hspX*. The perturbed parameter is ploted as a function of SA steps. In each panel, the five colored lines originated from different SA simulation. The horizontal dotted lines is for the base parameter value reported in Table 1.
(PDF)

S3 Fig Optimization profiles of kinetic parameters associated with *narK2-Rv1738*. The perturbed parameter is ploted as a function of SA steps. In each panel, the five colored lines originated from different SA simulation. The horizontal dotted lines is for the base parameter value reported in Table 1.
(PDF)

S4 Fig Scatter plots. The output (GFP in nM) as a function of synthesis and degradation rates of phosphorylated DevR (*k*_*srp*_ and *k*_*drp*_) and GFP (*k*_*sg*_ and *k*_*dg*_). Each panel consists of 10^3^ independent simulation data.
(PDF)

S5 Fig Sensitivity amplification of parameters related to *Rv3134c*. (PDF)

S6 Fig Sensitivity amplification of parameters related to *hspX*. (PDF)

S7 Fig Sensitivity amplification of parameters related to *narK2*. (PDF)

S8 Fig Sensitivity amplification of parameters related to *Rv1738*. (PDF)

S1 Table Kinetic parameters. List of all kinetic parameters used in the model.
(PDF)

S2 Table CC, RCC, PRCC values. Various correlation coefficient values are obtained by using 10% perturbation and 10^5^ indipendent run for synthesis and degradation rate parameters of four genes.
(PDF)

S3 Table CC, RCC, PRCC values. Various correlation coefficient values are obtained by using 10% perturbation and 10^5^ indipendent run for all the input parameter with output of *Rv3134c*.
(PDF)

S4 Table CC, RCC, PRCC values. Various correlation coefficient values are obtained by using 10% perturbation and 10^5^ indipendent run for all the input parameter with output of *hspX*.
(PDF)

S5 Table CC, RCC, PRCC values. Various correlation coefficient values are obtained by using 10% perturbation and 10^5^ indipendent run for all the input parameter with output of *narK2*.
(PDF)

S6 Table CC, RCC, PRCC values. Various correlation coefficient values are obtained by using 10% perturbation and 10^5^ indipendent run for all the input parameter with output of *Rv1738*.
(PDF)
