# Supplementary material for: Computational study of parameter sensitivity in DevR regulated gene expression
Source: PLoS One. 2020 Feb 13;15(2):e0228967. doi: 10.1371/journal.pone.0228967 (PMC7018068; doi:10.1371/journal.pone.0228967)
Supplement: S1 Text — Kinetic scheme and equations for phosphorylated DevR regulated gene expression. The text contains detailed scheme and differential equations for the model. (PDF) [file pone.0228967.s001.pdf]

Electronic Supplementary Information for Parameter sensitivity in DevR regulated gene expression

Jagannath Das,[1] Tarunendu Mapder,[1] Sudip Chattopadhyay,[1,*] and Suman K Banik[2,†]

[1] *Department of Chemistry, Indian Institute of Engineering Science and Technology, Shibpur, Howrah 711103, India*
[2] *Department of Chemistry, Bose Institute, 93/1 A P C Road, Kolkata 700009, India*

[*] sudip@chem.iiests.ac.in
[†] skbanik@jcbose.ac.in

KINETIC SCHEME FOR R_P REGULATED GENE EXPRESSION

Based on the experimental information the phosphorylated DevR regulated gene expression can be written as follows. In the following, P (S) and P^* (S^*) represent the inactive and active form of the promoters, respectively. $mGFP$ is the mRNA of GFP and GFP is the protein itself. While writing the promoter of a specific gene we use subscript, e.g., for *Rv3134c* we use P_{4c}, S_{4c}, etc.

A. Kinetics scheme for generation of R_P:

$$\xrightarrow{k_{srp}} Rp \xrightarrow{k_{drp}} \varnothing. \tag{S1}$$

B. Kinetic scheme for *Rv3134c*:

$$P_{4c} + R_p \underset{k_{u1}}{\overset{k_{b1}}{\rightleftharpoons}} P^*_{4c}, \tag{S2}$$

$$S_{4c} + R_p \underset{k_{u2}}{\overset{k_{b2}}{\rightleftharpoons}} S^*_{4c}, \tag{S3}$$

$$P^*_{4c} \xrightarrow{k_{sm1}} mGFP_{4c} + P^*_{4c}, \tag{S4}$$

$$S^*_{4c} \xrightarrow{k_{sm2}} mGFP_{4c} + S^*_{4c}, \tag{S5}$$

$$P^*_{4c}S^*_{4c} \xrightarrow{k_{sm3}} mGFP_{4c} + P^*_{4c}S^*_{4c}, \tag{S6}$$

$$mGFP_{4c} \xrightarrow{k_{dm}} \varnothing, \tag{S7}$$

$$mGFP_{4c} \xrightarrow{k_{sg}} mGFP_{4c} + GFP, \tag{S8}$$

$$GFP \xrightarrow{k_{dg}} \varnothing. \tag{S9}$$

C. Kinetic scheme for *hspX*:

$$P1_{px} + R_p \underset{k_{u3}}{\overset{k_{b3}}{\rightleftharpoons}} P1^*_{px}, \tag{S10}$$

$$P2_{px} + R_p \underset{k_{u4}}{\overset{k_{b4}}{\rightleftharpoons}} P2^*_{px}, \tag{S11}$$

$$S_{px} + R_p \underset{k_{u5}}{\overset{k_{b5}}{\rightleftharpoons}} S^*_{px}. \tag{S12}$$

$$P1^*_{px} \xrightarrow{k_{sm4}} mGFP_{px} + P1^*_{px}, \tag{S13}$$

$$P2^*_{px} \xrightarrow{k_{sm5}} mGFP_{px} + P2^*_{px}, \tag{S14}$$

$$S^*_{px} \xrightarrow{k_{sm6}} mGFP_{px} + S^*_{px}, \tag{S15}$$

$$P1^*_{px}P2^*_{px}S^*_{px} \xrightarrow{k_{sm7}} mGFP_{px} + P1^*_{px}P2^*_{px}S^*_{px}, \tag{S16}$$

$$mGFP_{px} \xrightarrow{k_{dm}} \varnothing, \tag{S17}$$

$$mGFP_{px} \xrightarrow{k_{sg}} mGFP_{px} + GFP, \tag{S18}$$

$$GFP \xrightarrow{k_{dg}} \varnothing. \tag{S19}$$

D. Kinetic scheme for *narK2-Rv1738*:

$$P1_{K2/38} + R_p \underset{k_{u6}}{\overset{k_{b6}}{\rightleftharpoons}} P1^*_{K2/38}, \tag{S20}$$

$$P2_{K2/38} + R_p \underset{k_{u7}}{\overset{k_{b7}}{\rightleftharpoons}} P2^*_{K2/38}, \tag{S21}$$

$$S1_{K2/38} + R_p \underset{k_{u8}}{\overset{k_{b8}}{\rightleftharpoons}} S1^*_{K2/38}, \tag{S22}$$

$$S2_{K2/38} + R_p \underset{k_{u9}}{\overset{k_{b9}}{\rightleftharpoons}} S2^*_{K2/38}. \tag{S23}$$

$$P1^*_{K2/38} \xrightarrow{k_{sm8}} mGFP_{K2} + P1^*_{K2/38}, \tag{S24}$$

$$P1^*_{K2/38} \xrightarrow{k_{sm9}} mGFP_{38} + P1^*_{K2/38}, \tag{S25}$$

$$P2^*_{K2/38} \xrightarrow{k_{sm10}} mGFP_{K2} + P2^*_{K2/38}, \tag{S26}$$

$$P2^*_{K2/38} \xrightarrow{k_{sm11}} mGFP_{38} + P2^*_{K2/38}, \tag{S27}$$

$$S1^*_{K2/38} \xrightarrow{k_{sm12}} mGFP_{K2} + S1^*_{K2/38}, \tag{S28}$$

$$S1^*_{K2/38} \xrightarrow{k_{sm13}} mGFP_{38} + S1^*_{K2/38}, \tag{S29}$$

$$S2^*_{K2/38} \xrightarrow{k_{sm14}} mGFP_{K2} + S2^*_{K2/38}, \tag{S30}$$

$$S2^*_{K2/38} \xrightarrow{k_{sm15}} mGFP_{38} + S2^*_{K2/38}, \tag{S31}$$

$$P1^*_{K2/38}S1^* \xrightarrow{k_{sm16}} mGFP_{K2} + P1^*_{K2/38}S1^*, \tag{S32}$$

$$P1^*_{K2/38}S1^* \xrightarrow{k_{sm17}} mGFP_{38} + P1^*_{K2/38}S1^*, \tag{S33}$$

$$P2^*_{K2/38}S2^* \xrightarrow{k_{sm18}} mGFP_{K2} + P2^*_{K2/38}S2^*, \tag{S34}$$

$$P2^*_{K2/38}S2^* \xrightarrow{k_{sm19}} mGFP_{38} + P2^*_{K2/38}S2^*. \tag{S35}$$

$$mGFP_{K2} \xrightarrow{k_{dm}} \varnothing, \tag{S36}$$

$$mGFP_{38} \xrightarrow{k_{dm}} \varnothing, \tag{S37}$$

$$mGFP_{K2} \xrightarrow{k_{sg}} mGFP_{K2} + GFP, \tag{S38}$$

$$mGFP_{38} \xrightarrow{k_{sg}} mGFP_{38} + GFP, \tag{S39}$$

$$GFP \xrightarrow{k_{dg}} \varnothing. \tag{S40}$$

KINETIC EQUATIONS

Based on the kinetic scheme given in Eqs. (S1-S40) we write the kinetic equations of the model. We note here that, while creating the scatter plots and subsequent calculation of correlation coefficients we make use of the following set of ordinary differential equations.

DevR:

$$\frac{d[R_p]}{dt} = k_{srp} - k_{drp}[R_p]. \tag{S41}$$

Rv3134c:

$$\frac{d[P_{4c}^*]}{dt} = k_{b1}[P_{4c}][R_p] - k_{u1}[P_{4c}^*], \tag{S42}$$

$$\frac{d[S_{4c}^*]}{dt} = k_{b2}[S_{4c}][R_p] - k_{u2}[S_{4c}^*], \tag{S43}$$

$$\begin{aligned}\frac{d[mGFP_{4c}]}{dt} &= k_{sm1}[P_{4c}^*] + k_{sm2}[S_{4c}^*] + k_{sm3}[P_{4c}^*][S_{4c}^*] \\ &\quad -k_{dm}[mGFP_{4c}],\end{aligned} \tag{S44}$$

$$\frac{d[GFP]}{dt} = k_{sg}[mGFP_{4c}] - k_{dg}[GFP]. \tag{S45}$$

hspX:

$$\frac{d[P1_{px}^*]}{dt} = k_{b3}[P1_{px}][R_p] - k_{u3}[P1_{px}^*], \tag{S46}$$

$$\frac{d[P2_{px}^*]}{dt} = k_{b4}[P2_{px}][R_p] - k_{u4}[P2_{px}^*], \tag{S47}$$

$$\frac{d[S_{px}^*]}{dt} = k_{b5}[S_{px}][R_p] - k_{u5}[S_{px}^*], \tag{S48}$$

$$\begin{aligned}\frac{d[mGFP_{px}]}{dt} &= k_{sm4}[P1_{px}^*] + k_{sm5}[P2_{px}^*] + k_{sm6}[S_{px}^*] \\ &\quad +k_{sm7}[P1_{px}^*][P2_{px}^*][S_{px}^*] \\ &\quad -k_{dm}[mGFP_{px}],\end{aligned} \tag{S49}$$

$$\frac{d[GFP]}{dt} = k_{sg}[mGFP_{px}] - k_{dg}[GFP]. \tag{S50}$$

narK2-Rv1738:

$$\frac{d[P1_{K2/38}^*]}{dt} = k_{b6}[P1_{K2/38}][R_p] - k_{u6}[P1_{K2/38}^*], \tag{S51}$$

$$\frac{d[P2_{K2/38}^*]}{dt} = k_{b7}[P2_{K2/38}][R_p] - k_{u7}[P2_{K2/38}^*], \tag{S52}$$

$$\frac{d[S1_{K2/38}^*]}{dt} = k_{b8}[S1_{K2/38}][R_p] - k_{u8}[S1_{K2/38}^*], \tag{S53}$$

$$\frac{d[S2_{K2/38}^*]}{dt} = k_{b9}[S2_{K2/38}][R_p] - k_{u9}[S2_{K2/38}^*], \tag{S54}$$

$$\begin{aligned}\frac{d[mGFP_{K2}]}{dt} &= k_{sm8}[P1_{K2/38}^*] + k_{sm10}[P2_{K2/38}^*] \\ &\quad +k_{sm12}[S1_{K2/38}^*] + k_{sm14}[S2_{K2/38}^*] \\ &\quad +k_{sm16}[P1_{K2/38}^*][S1_{K2/38}^*] \\ &\quad +k_{sm18}[P2_{K2/38}^*][S2_{K2/38}^*] \\ &\quad -k_{dm}[mGFP_{K2}],\end{aligned} \tag{S55}$$

$$\frac{d[GFP]}{dt} = k_{sg}[mGFP_{K2}] - k_{dg}[GFP], \tag{S56}$$

$$\begin{aligned}\frac{d[mGFP_{38}]}{dt} &= k_{sm9}[P1_{K2/38}^*] + k_{sm11}[P2_{K2/38}^*] \\ &\quad +k_{sm13}[S1_{K2/38}^*] + k_{sm15}[S2_{K2/38}^*] \\ &\quad +k_{sm17}[P1_{K2/38}^*][S1_{K2/38}^*] \\ &\quad +k_{sm19}[P2_{K2/38}^*][S2_{K2/38}^*] \\ &\quad -k_{dm}[mGFP_{38}],\end{aligned} \tag{S57}$$

$$\frac{d[GFP]}{dt} = k_{sg}[mGFP_{38}] - k_{dg}[GFP]. \tag{S58}$$

INITIAL CONDITIONS IN STOCHASTIC OPTIMIZATION

To carry out stochastic optimization we implemented random initial values of the model parameters. To this end, we used two sets of random initial conditions. In one set, parameters are randomly generated from the range $10^{-7} - 10^{-5}$. In the second set, same was done using the range $10^{-5} - 10^{-3}$. We note that initial conditions lower than 10^{-7} and higher than 10^{-3} are unable to reproduce desired experimental profiles.
