# Supplementary figures and images for: Computational study of parameter sensitivity in DevR regulated gene expression
Source: PLoS One. 2020 Feb 13;15(2):e0228967. doi: 10.1371/journal.pone.0228967 (PMC7018068; doi:10.1371/journal.pone.0228967)

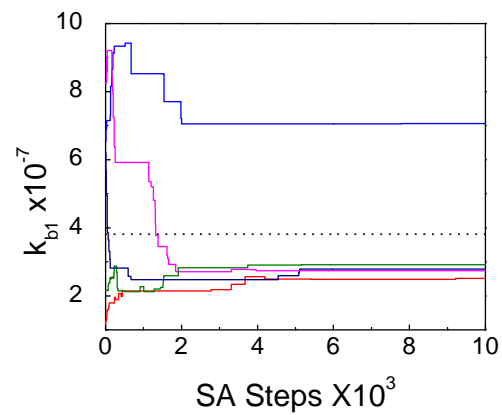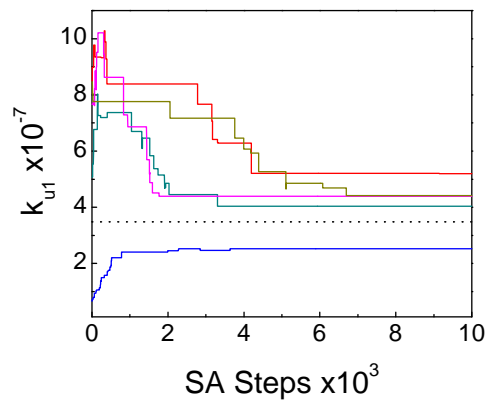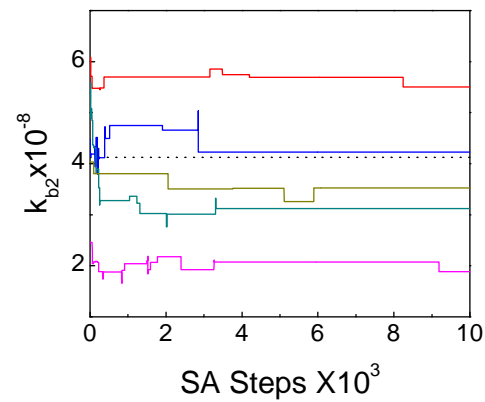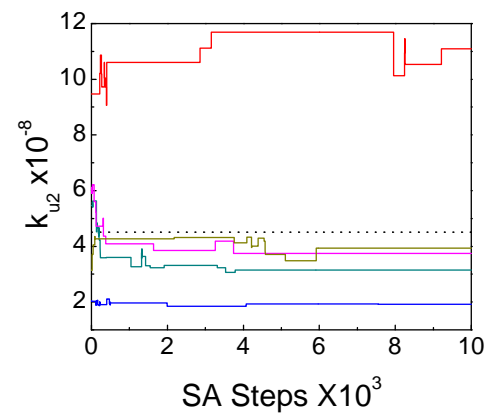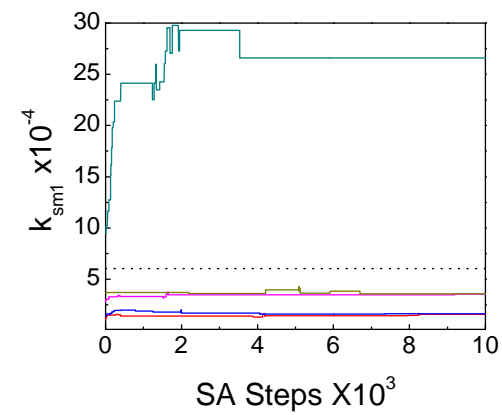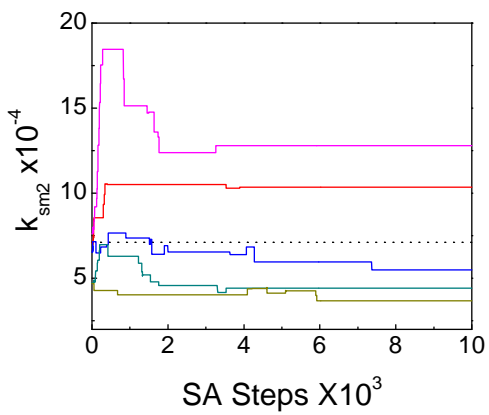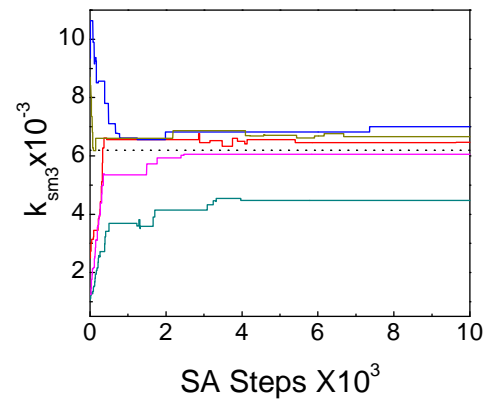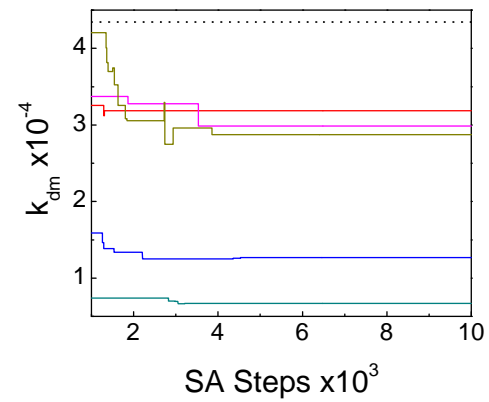

Supplement: S1 Fig — The perturbed parameter is ploted as a function of SA steps. In each panel, the five colored lines originated from different SA simulation. The horizontal dotted lines is for the base parameter value reported in Table 1. (PDF) [file pone.0228967.s002.pdf]

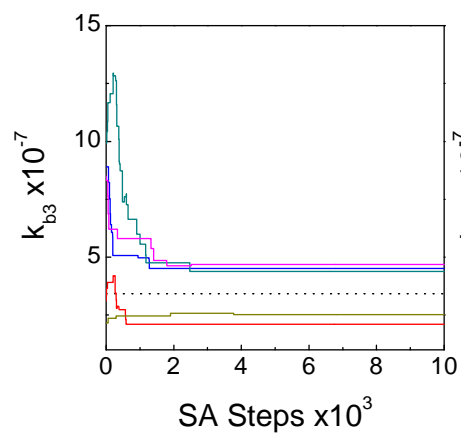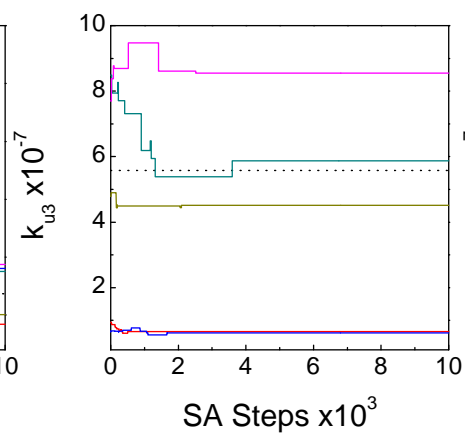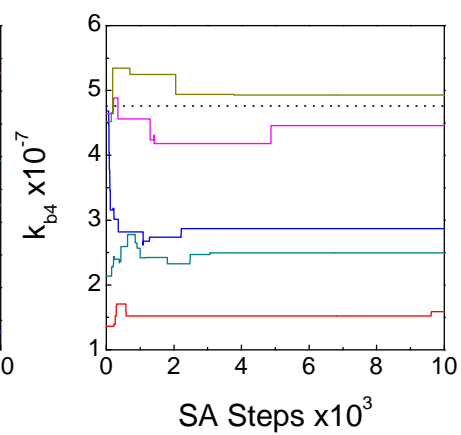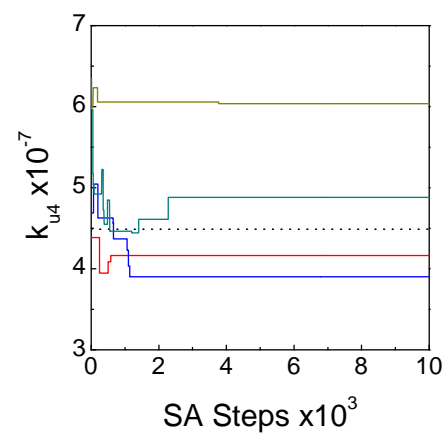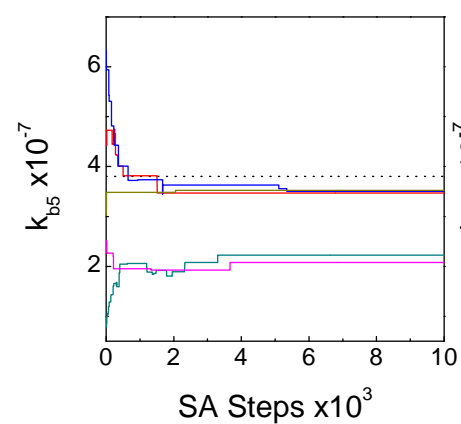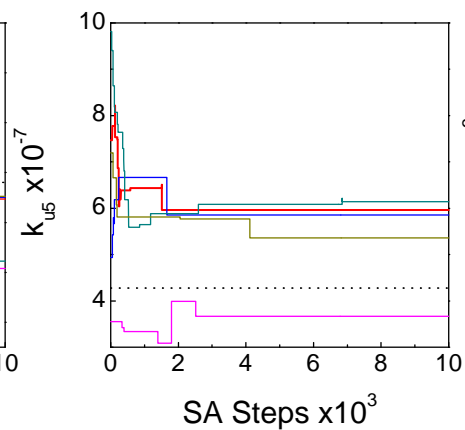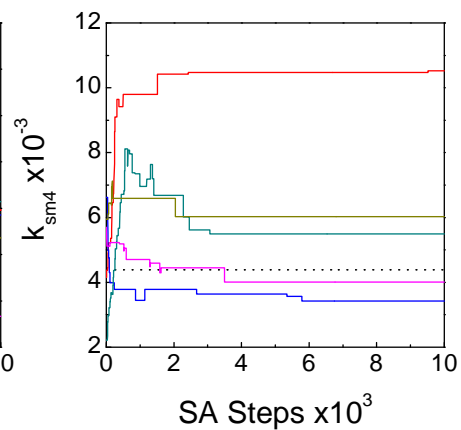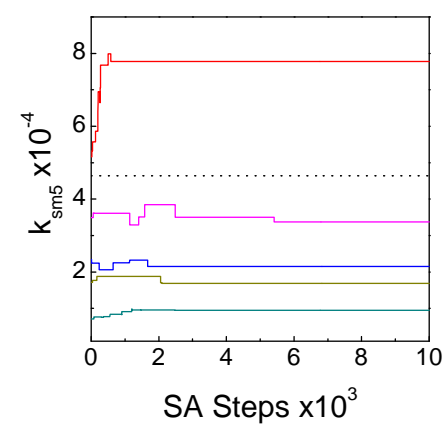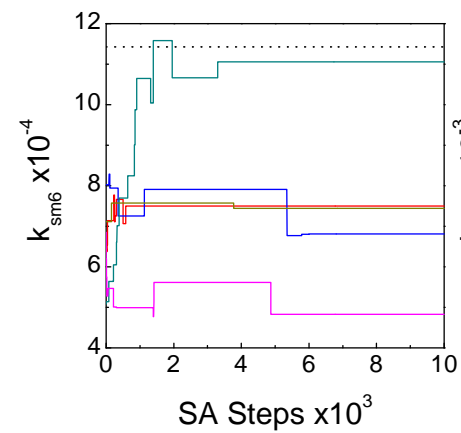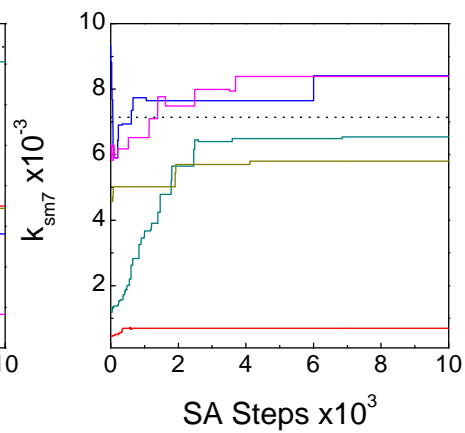

Supplement: S2 Fig — The perturbed parameter is ploted as a function of SA steps. In each panel, the five colored lines originated from different SA simulation. The horizontal dotted lines is for the base parameter value reported in Table 1. (PDF) [file pone.0228967.s003.pdf]

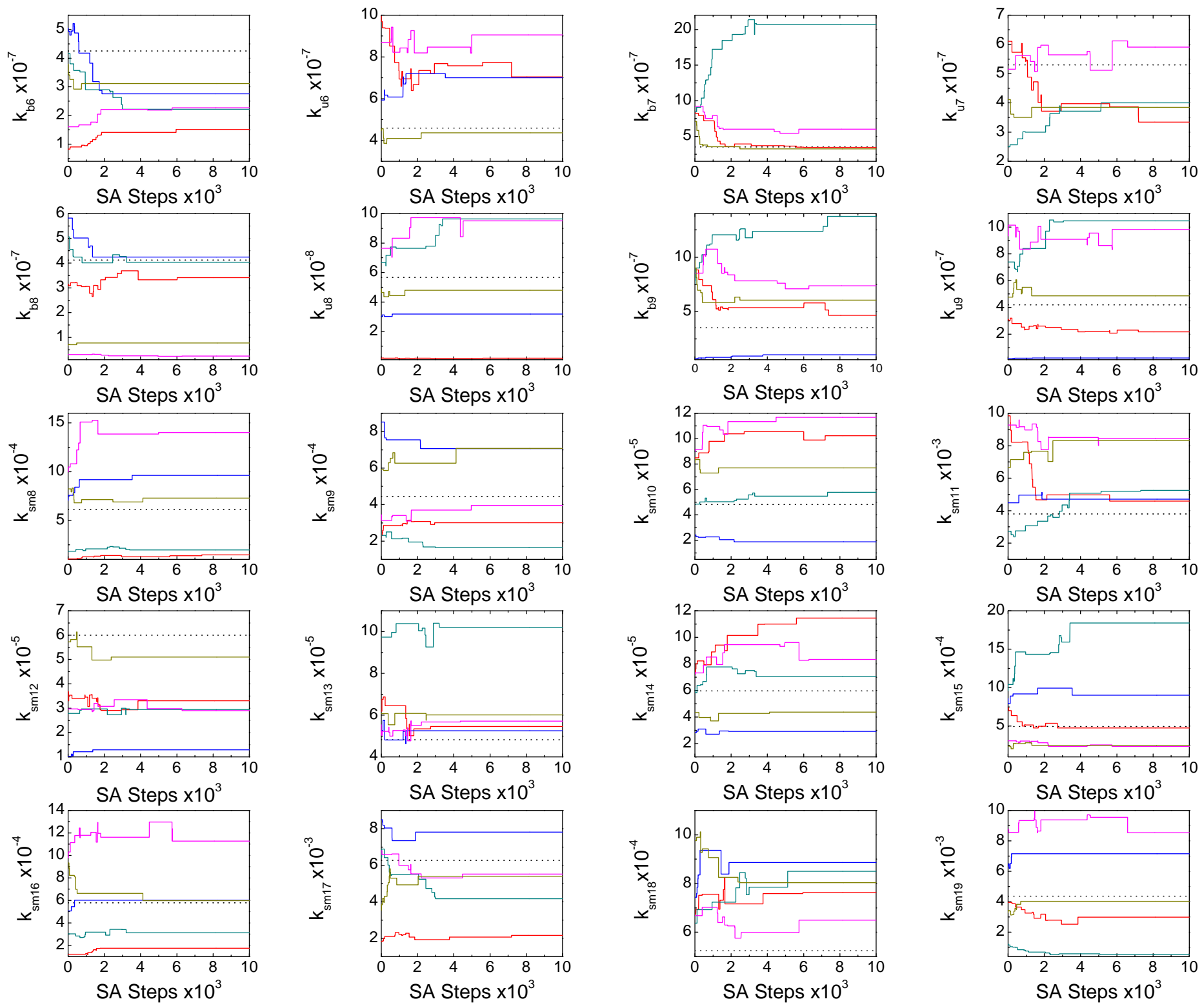

Supplement: S3 Fig — The perturbed parameter is ploted as a function of SA steps. In each panel, the five colored lines originated from different SA simulation. The horizontal dotted lines is for the base parameter value reported in Table 1. (PDF) [file pone.0228967.s004.pdf]

*Rv3134c*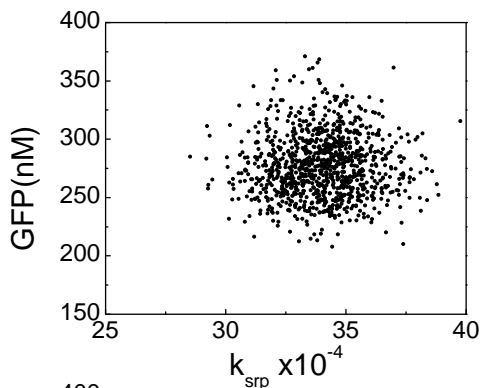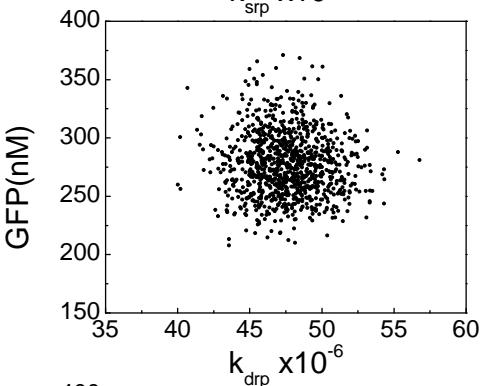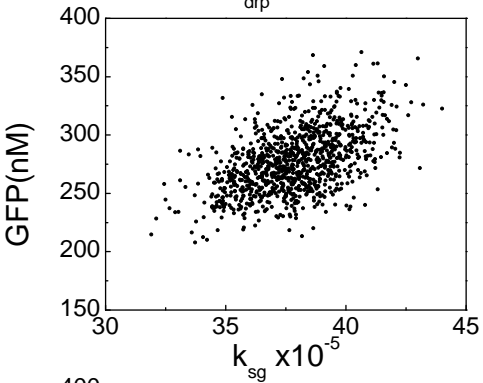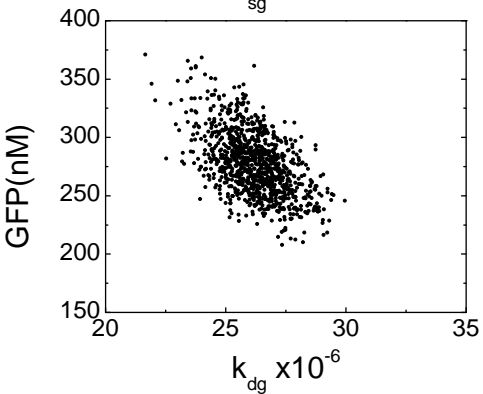*hspX*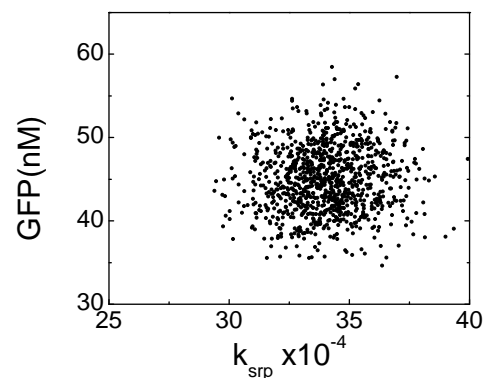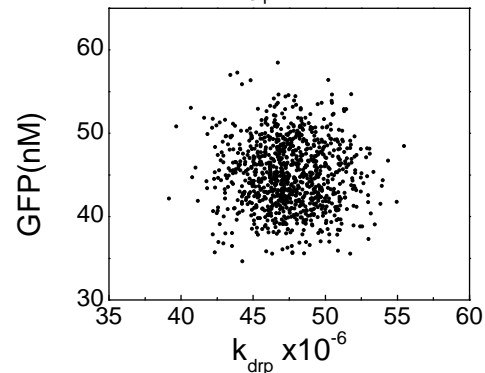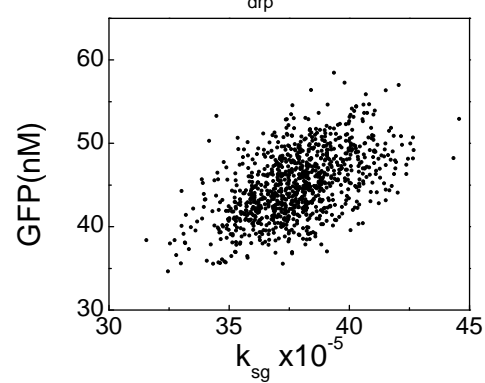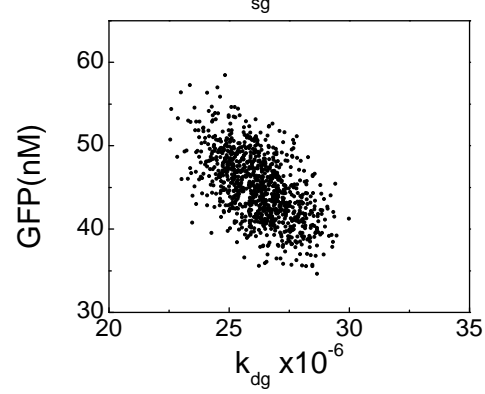*narK2*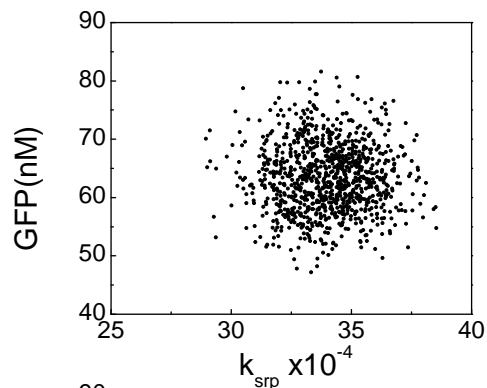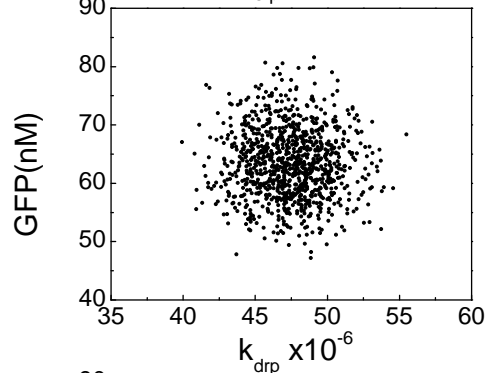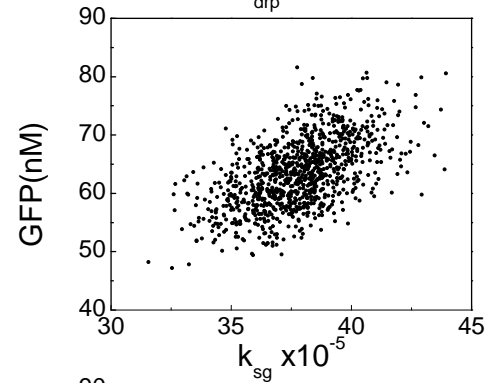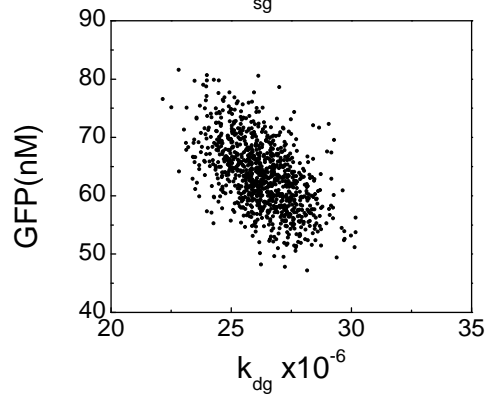*Rv1738*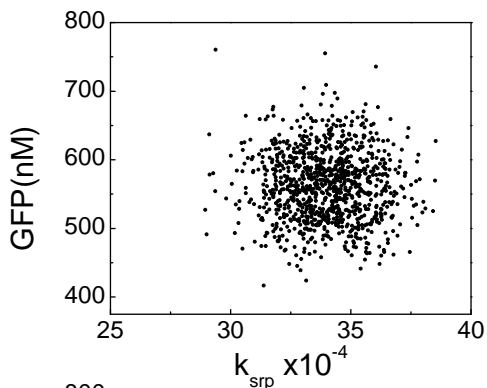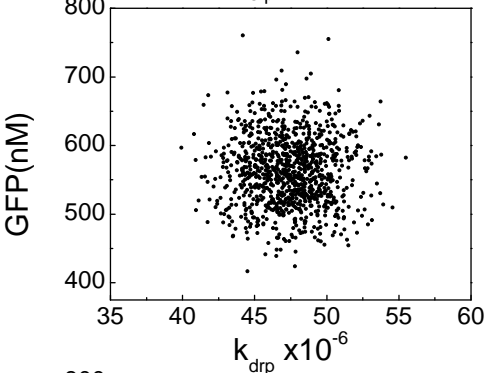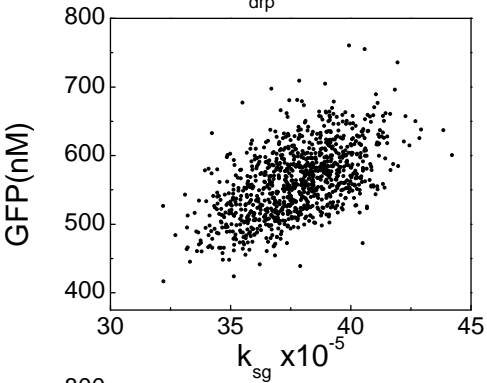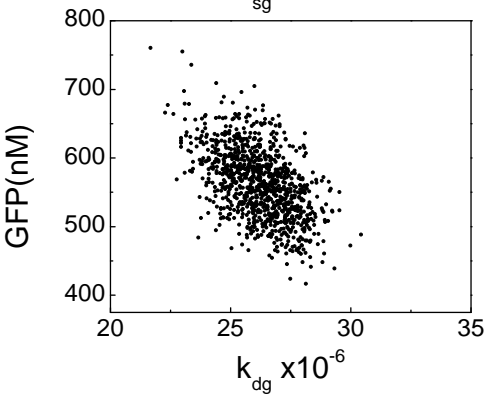

Supplement: S4 Fig — The output (GFP in nM) as a function of synthesis and degradation rates of phosphorylated DevR (ksrp and kdrp) and GFP (ksg and kdg). Each panel consists of 103 independent simulation data. (PDF) [file pone.0228967.s005.pdf]

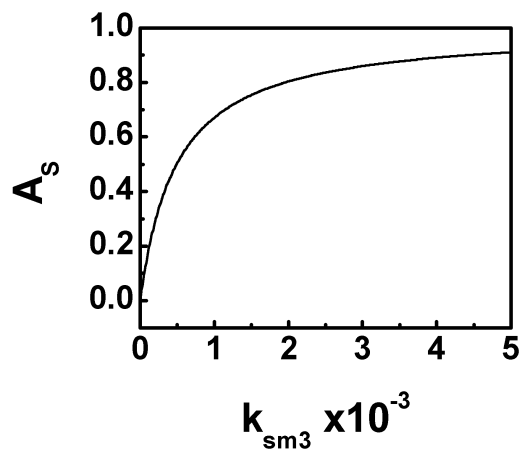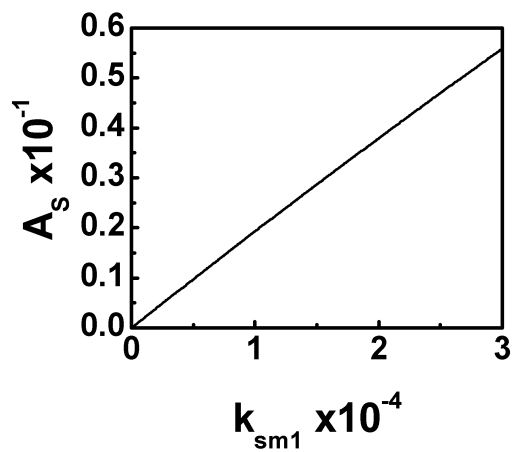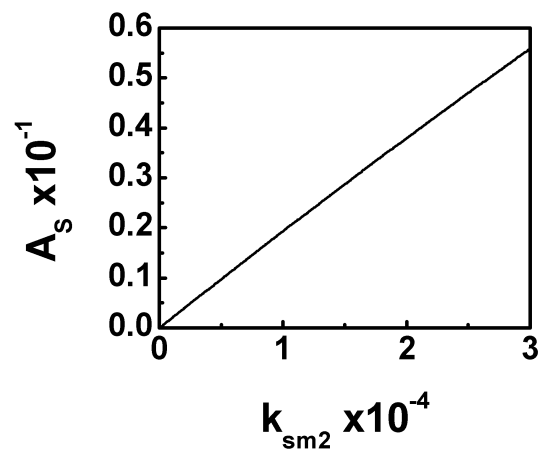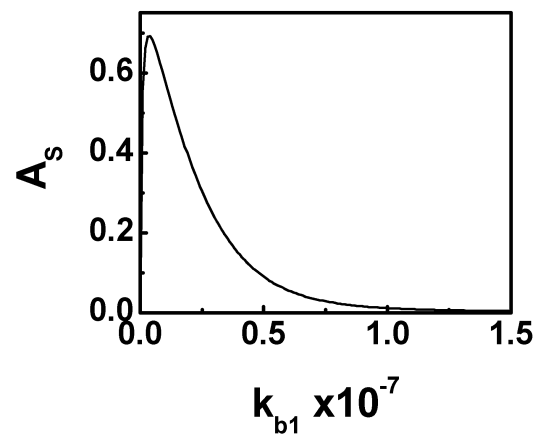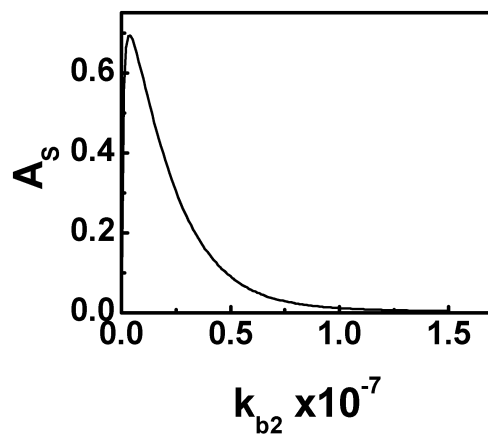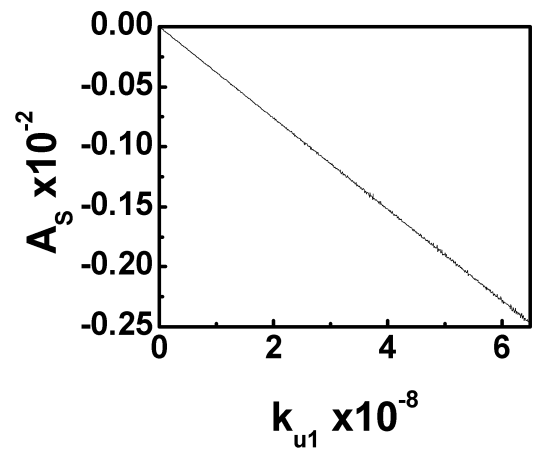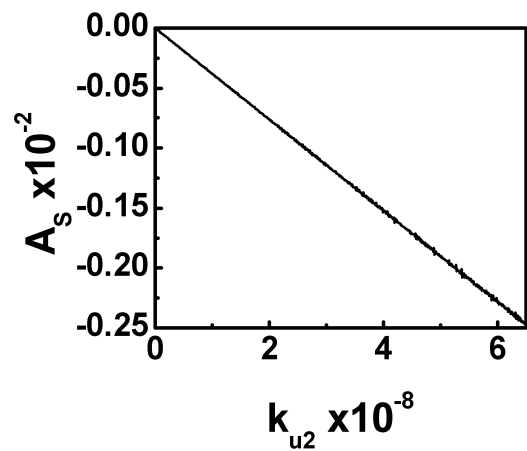

Supplement: S5 Fig — (PDF) [file pone.0228967.s006.pdf]

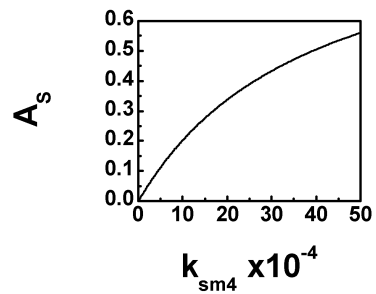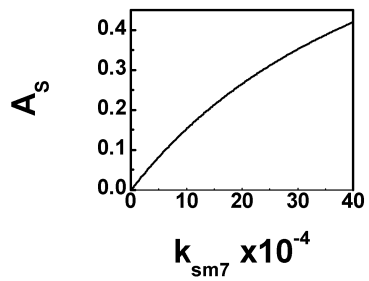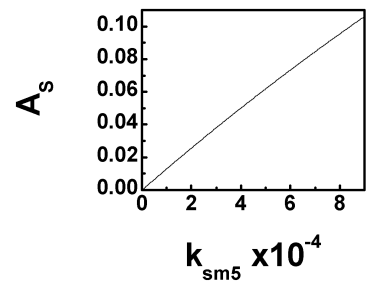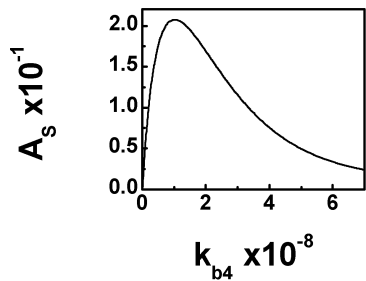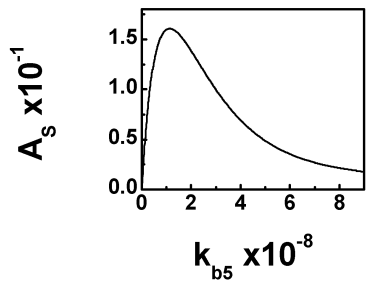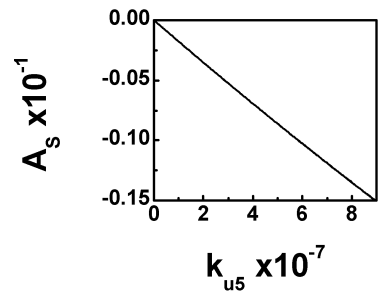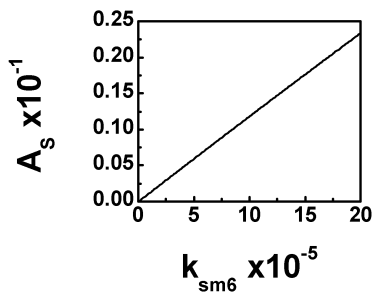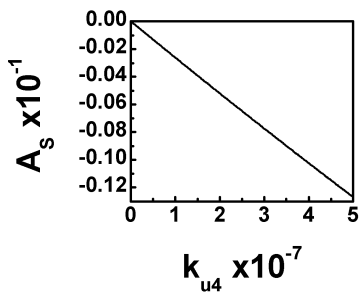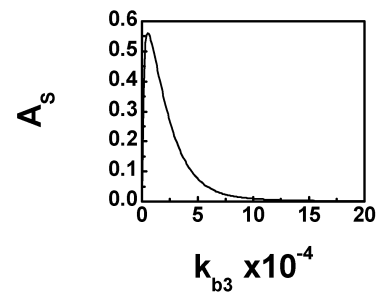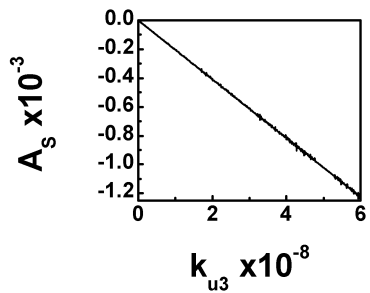

Supplement: S6 Fig — (PDF) [file pone.0228967.s007.pdf]

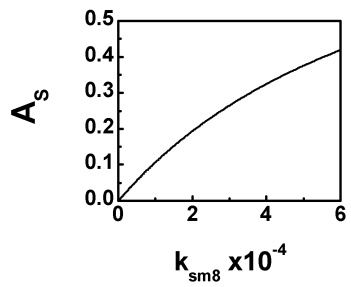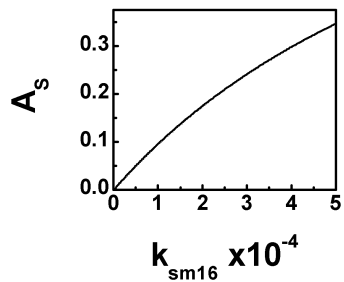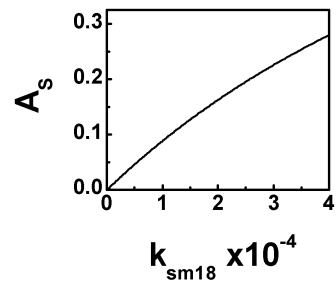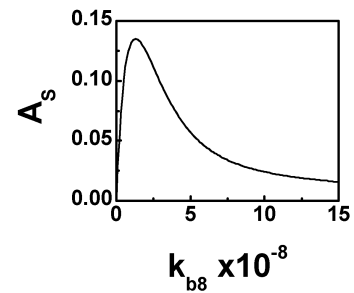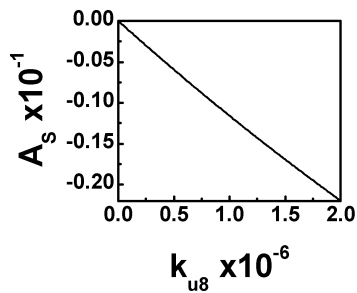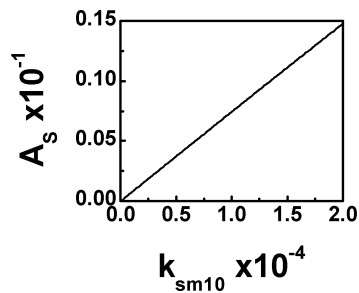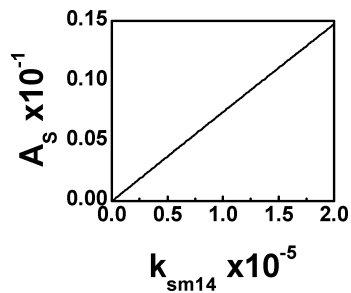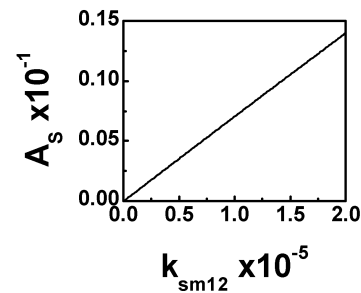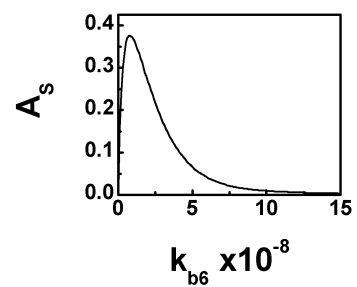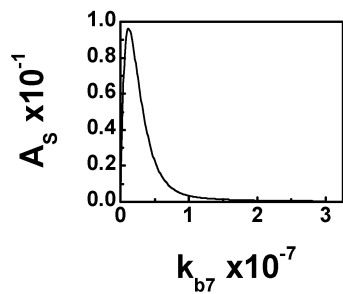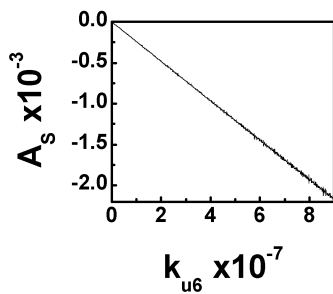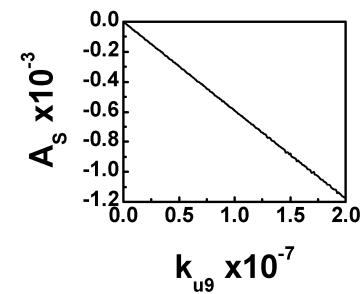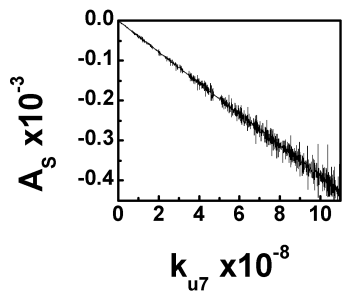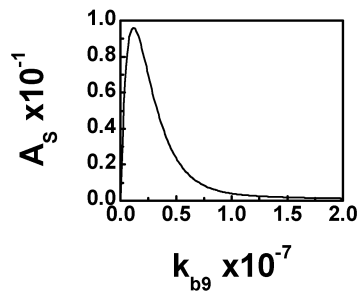

Supplement: S7 Fig — (PDF) [file pone.0228967.s008.pdf]

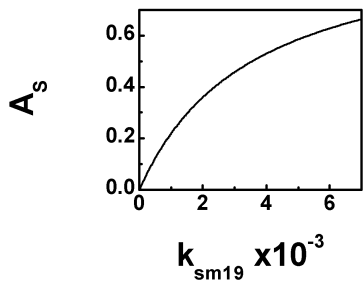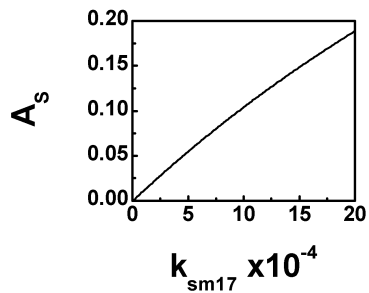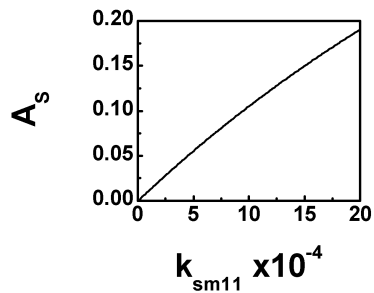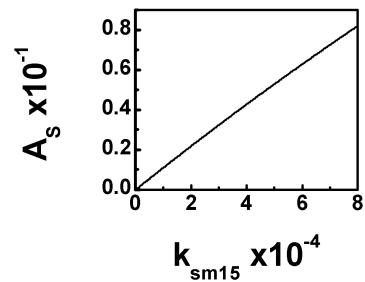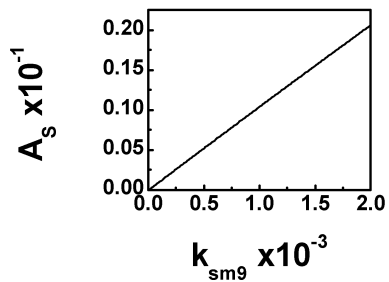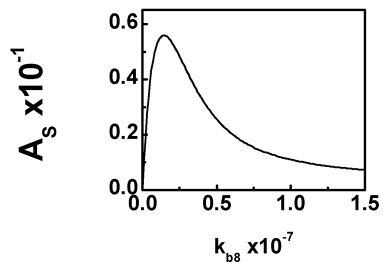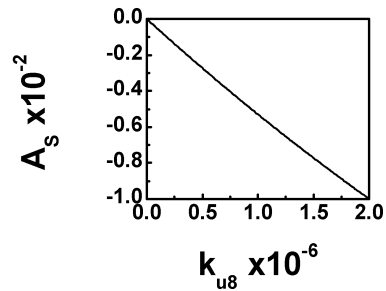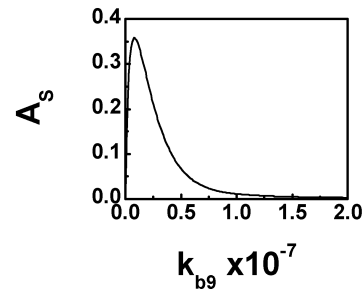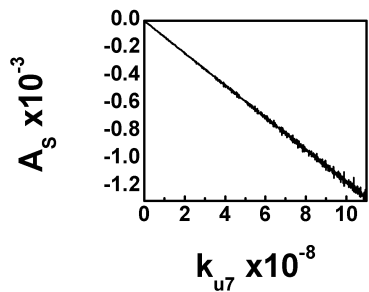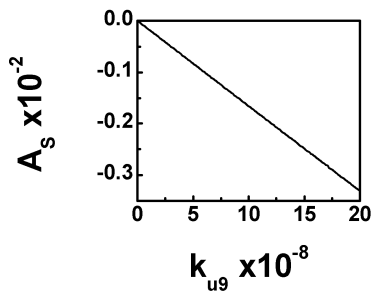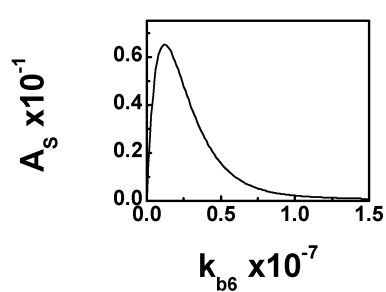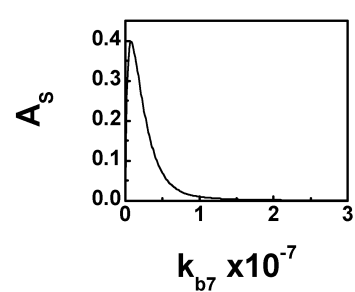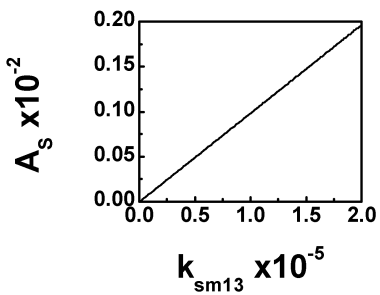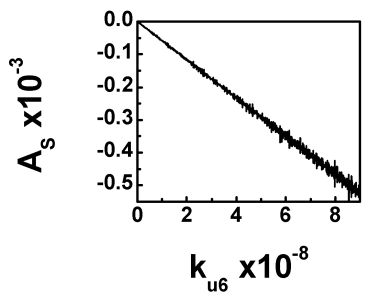

Supplement: S8 Fig — (PDF) [file pone.0228967.s009.pdf]
