# Supplementary material for: Computational study of parameter sensitivity in DevR regulated gene expression
Source: PLoS One. 2020 Feb 13;15(2):e0228967. doi: 10.1371/journal.pone.0228967 (PMC7018068; doi:10.1371/journal.pone.0228967)
Supplement: S1 Table — List of all kinetic parameters used in the model. (PDF) [file pone.0228967.s010.pdf]

S1 Table. List of kinetic parameters used in the model

Parameter	Description
k_{srp}	Synthesis rate of R_p
k_{drp}	Degradation rate of R_p
k_{b1}	Activation rate of P_{4c} to P^*_{4c}
k_{u1}	Deactivation rate of P^*_{4c} to P_{4c}
k_{b2}	Activation rate of S_{4c} to S^*_{4c}
k_{u2}	Deactivation rate of S^*_{4c} to S_{4c}
k_{b3}	Activation rate of $P1_{px}$ to $P1^*_{px}$
k_{u3}	Deactivation rate of $P1^*_{px}$ to $P1_{px}$
k_{b4}	Activation rate of $P2_{px}$ to $P2^*_{px}$
k_{u4}	Deactivation rate of $P2^*_{px}$ to $P2_{px}$
k_{b5}	Activation rate of S_{px} to S^*_{px}
k_{u5}	Deactivation rate of S^*_{px} to S_{px}
k_{b6}	Activation rate of $P1_{K2/38}$ to $P1^*_{K2/38}$
k_{u6}	Deactivation rate of $P1^*_{K2/38}$ to $P1_{K2/38}$
k_{b7}	Activation rate of $P2_{K2/38}$ to $P2^*_{K2/38}$
k_{u7}	Deactivation rate of $P2^*_{K2/38}$ to $P2_{K2/38}$
k_{b8}	Activation rate of $S1_{K2/38}$ to $S1^*_{K2/38}$
k_{u8}	Deactivation rate of $S1^*_{K2/38}$ to $S1_{K2/38}$
k_{b9}	Activation rate of $S2_{K2/38}$ to $S2^*_{K2/38}$
k_{u9}	Deactivation rate of $S2^*_{K2/38}$ to $S2_{K2/38}$
k_{sm1}	Synthesis rate of $mGFP_{4c}$ to P^*_{4c}
k_{sm2}	Synthesis rate of $mGFP_{4c}$ to S^*_{4c}
k_{sm3}	Synthesis rate of $mGFP_{4c}$ to $P^*_{4c}{S_{4c}}^*$
k_{sm4}	Synthesis rate of $mGFP_{px}$ to $P1^*_{px}$
k_{sm5}	Synthesis rate of $mGFP_{px}$ to $P2^*_{px}$
k_{sm6}	Synthesis rate of $mGFP_{px}$ to S^*_{px}
k_{sm7}	Synthesis rate of $mGFP_{px}$ to $P1^*_{px}P2^*_{px}S^*_{px}$
k_{sm8}	Synthesis rate of $mGFP_{K2}$ to $P1^*_{K2/38}$
k_{sm9}	Synthesis rate of $mGFP_{38}$ to $P1^*_{K2/38}$
k_{sm10}	Synthesis rate of $mGFP_{K2}$ to $P2^*_{K2/38}$
k_{sm11}	Synthesis rate of $mGFP_{38}$ to $P2^*_{K2/38}$
k_{sm12}	Synthesis rate of $mGFP_{K2}$ to $S1^*_{K2/38}$
k_{sm13}	Synthesis rate of $mGFP_{38}$ to $S1^*_{K2/38}$
k_{sm14}	Synthesis rate of $mGFP_{K2}$ to $S2^*_{K2/38}$
k_{sm15}	Synthesis rate of $mGFP_{38}$ to $S2^*_{K2/38}$
k_{sm16}	Synthesis rate of $mGFP_{K2}$ to $P1^*_{K2/38}S1^*_{K2/38}$
k_{sm17}	Synthesis rate of $mGFP_{38}$ to $P1^*_{K2/38}S1^*_{K2/38}$
k_{sm18}	Synthesis rate of $mGFP_{K2}$ to $P2^*_{K2/38}S2^*_{K2/38}$
k_{sm19}	Synthesis rate of $mGFP_{38}$ to $P2^*_{K2/38}S2^*_{K2/38}$
k_{dm}	Degradation rate of all $mGFP$
k_{sg}	Synthesis rate of GFP
k_{dg}	Degradation rate of GFP
