# Supplementary material for: Computational study of parameter sensitivity in DevR regulated gene expression
Source: PLoS One. 2020 Feb 13;15(2):e0228967. doi: 10.1371/journal.pone.0228967 (PMC7018068; doi:10.1371/journal.pone.0228967)
Supplement: S2 Table — Various correlation coefficient values are obtained by using 10% perturbation and 105 indipendent run for synthesis and degradation rate parameters of four genes. (PDF) [file pone.0228967.s011.pdf]

S2 Table. CC, RCC, PRCC values for synthesis and degradation rate parameters of four genes using 10% perturbation.

		Rv3134c			*hspX*			*narK2*			*Rv1738*		
	Parameter	CC	RCC	PRCC	CC	RCC	PRCC	CC	RCC	PRCC	CC	RCC	PRCC
Set 1	k_{srp}	0.012	0.012	0.034	0.026	0.025	0.101	0.019	0.019	0.079	0.025	0.025	0.098
	k_{drp}	-0.011	-0.011	-0.034	-0.027	-0.026	-0.099	-0.023	-0.021	-0.086	-0.026	-0.027	-0.100
	k_{sg}	0.503	0.495	0.881	0.531	0.527	0.888	0.541	0.534	0.896	0.533	0.529	0.894
	k_{dg}	-0.517	-0.503	-0.881	-0.539	-0.522	-0.888	-0.551	-0.537	-0.896	-0.545	-0.529	-0.893
Set 2	k_{srp}	0.009	0.009	0.017	0.026	0.025	0.089	0.014	0.012	0.069	0.020	0.019	0.071
	k_{drp}	0.000	-0.001	-0.022	-0.021	-0.019	-0.075	-0.011	-0.009	-0.064	-0.017	-0.016	-0.070
	k_{sg}	0.514	0.509	0.884	0.533	0.529	0.893	0.537	0.533	0.895	0.533	0.525	0.893
	k_{dg}	-0.520	-0.502	-0.882	-0.543	-0.526	-0.893	-0.547	-0.530	-0.894	-0.545	-0.528	-0.893
Mean	k_{srp}	0.011	0.011	0.026	0.026	0.025	0.095	0.017	0.016	0.074	0.023	0.022	0.085
	k_{drp}	-0.006	-0.006	-0.028	-0.024	-0.023	-0.087	-0.017	-0.015	-0.075	-0.022	-0.022	-0.085
	k_{sg}	0.509	0.502	0.883	0.532	0.528	0.891	0.539	0.534	0.896	0.533	0.527	0.894
	k_{dg}	-0.519	-0.503	-0.882	-0.541	-0.524	-0.891	-0.549	-0.534	-0.895	-0.545	-0.529	-0.893
