# Supplementary material for: Computational study of parameter sensitivity in DevR regulated gene expression
Source: PLoS One. 2020 Feb 13;15(2):e0228967. doi: 10.1371/journal.pone.0228967 (PMC7018068; doi:10.1371/journal.pone.0228967)
Supplement: S3 Table — Various correlation coefficient values are obtained by using 10% perturbation and 105 indipendent run for all the input parameter with output of Rv3134c. (PDF) [file pone.0228967.s012.pdf]

S3 Table. CC, RCC, PRCC values for all the input parameter with output (GFP concentration) of *Rv3134c* using 10% perturbation.

	CC			RCC			PRCC		
Parameter	Set1	Set2	Mean	Set1	Set2	Mean	Set1	Set2	Mean
k_{dm}	-0.520	-0.524	-0.522	-0.501	-0.506	-0.504	-0.882	-0.883	-0.883
k_{sm3}	0.420	0.401	0.411	0.413	0.394	0.404	0.837	0.824	0.831
k_{sm1}	0.040	0.089	0.065	0.039	0.087	0.063	0.145	0.299	0.222
k_{sm2}	0.053	0.031	0.042	0.049	0.030	0.400	0.169	0.105	0.137
k_{b2}	0.008	0.003	0.006	0.007	0.004	0.006	0.024	0.014	0.019
k_{b1}	0.005	0.005	0.005	0.003	0.004	0.004	0.008	0.017	0.013
k_{u2}	-0.002	-0.004	-0.003	-0.001	-0.006	-0.004	-0.005	-0.012	-0.009
k_{u1}	0.004	0.001	0.003	0.002	0.001	0.002	-0.002	-0.002	-0.002
