# Supplementary material for: Computational study of parameter sensitivity in DevR regulated gene expression
Source: PLoS One. 2020 Feb 13;15(2):e0228967. doi: 10.1371/journal.pone.0228967 (PMC7018068; doi:10.1371/journal.pone.0228967)
Supplement: S4 Table — Various correlation coefficient values are obtained by using 10% perturbation and 105 indipendent run for all the input parameter with output of hspX. (PDF) [file pone.0228967.s013.pdf]

S4 Table. CC, RCC, PRCC values for all the input parameter with output (GFP concentration) of *hspX* using 10% perturbation.

	CC			RCC			PRCC		
Parameter	Set1	Set2	Mean	Set1	Set2	Mean	Set1	Set2	Mean
k_{dm}	-0.540	-0.543	-0.542	-0.523	-0.527	-0.525	-0.888	-0.892	-0.890
k_{sm4}	0.281	0.220	0.251	0.275	0.213	0.244	0.710	0.628	0.669
k_{sm7}	0.182	0.221	0.202	0.178	0.216	0.197	0.547	0.621	0.584
k_{sm5}	0.040	0.047	0.044	0.045	0.045	0.045	0.167	0.144	0.156
k_{sm6}	0.015	0.059	0.037	0.014	0.056	0.035	0.061	0.193	0.077
k_{b3}	0.015	0.010	0.013	0.015	0.011	0.013	0.052	0.047	0.050
k_{u3}	-0.015	-0.006	-0.011	-0.013	-0.006	-0.010	-0.048	-0.034	-0.041
k_{b5}	0.005	0.002	0.004	0.005	0.001	0.003	0.027	0.019	0.023
k_{u4}	-0.001	-0.005	-0.003	-0.001	-0.003	-0.002	-0.016	-0.023	-0.020
k_{u5}	-0.009	-0.006	-0.008	-0.010	-0.007	-0.009	-0.019	-0.018	-0.019
k_{b4}	0.008	0.012	0.010	0.008	0.010	0.009	0.018	0.020	0.019
