# Supplementary material for: Computational study of parameter sensitivity in DevR regulated gene expression
Source: PLoS One. 2020 Feb 13;15(2):e0228967. doi: 10.1371/journal.pone.0228967 (PMC7018068; doi:10.1371/journal.pone.0228967)
Supplement: S5 Table — Various correlation coefficient values are obtained by using 10% perturbation and 105 indipendent run for all the input parameter with output of narK2. (PDF) [file pone.0228967.s014.pdf]

S5 Table. CC, RCC, PRCC values for all the input parameter with output (GFP concentration) of *narK2* using 10% perturbation.

	CC			RCC			PRCC		
Parameter	Set1	Set2	Mean	Set1	Set2	Mean	Set1	Set2	Mean
k_{dm}	-0.550	-0.548	-0.549	-0.534	-0.531	-0.533	-0.896	-0.895	-0.896
k_{sm8}	0.152	0.231	0.192	0.146	0.227	0.187	0.478	0.643	0.561
k_{sm16}	0.177	0.124	0.151	0.172	0.121	0.147	0.549	0.419	0.484
k_{sm18}	0.159	0.137	0.148	0.155	0.132	0.139	0.517	0.432	0.475
k_{sm14}	0.013	0.026	0.020	0.011	0.027	0.019	0.063	0.076	0.070
k_{sm12}	0.019	0.016	0.018	0.018	0.016	0.017	0.066	0.068	0.067
k_{sm10}	0.010	0.011	0.011	0.009	0.010	0.010	0.053	0.046	0.050
k_{b6}	0.011	0.008	0.010	0.010	0.008	0.009	0.018	0.036	0.027
k_{b7}	-0.003	-0.011	-0.007	0.002	-0.011	-0.005	-0.011	-0.030	-0.021
k_{u6}	-0.012	-0.003	-0.008	-0.012	-0.004	-0.008	-0.021	-0.011	-0.016
k_{u7}	0.002	-0.001	0.001	0.002	-0.001	0.001	0.018	0.014	0.016
k_{b9}	0.005	0.001	0.003	0.007	0.002	0.005	0.023	0.007	0.015
k_{u8}	-0.001	-0.007	-0.004	-0.002	-0.004	-0.003	-0.012	-0.010	-0.011
k_{b8}	-0.001	-0.002	-0.002	-0.001	-0.002	-0.002	-0.012	-0.007	-0.010
k_{u9}	-0.001	-0.002	-0.001	-0.001	-0.002	-0.002	-0.012	-0.007	-0.020
