# Supplementary material for: Computational study of parameter sensitivity in DevR regulated gene expression
Source: PLoS One. 2020 Feb 13;15(2):e0228967. doi: 10.1371/journal.pone.0228967 (PMC7018068; doi:10.1371/journal.pone.0228967)
Supplement: S6 Table — Various correlation coefficient values are obtained by using 10% perturbation and 105 indipendent run for all the input parameter with output of Rv1738. (PDF) [file pone.0228967.s015.pdf]

S6 Table. CC, RCC, PRCC values for all the Input parameter with output (GFP concentration) of *Rv1738* using 10% perturbation.

	CC			RCC			PRCC		
Parameter	Set1	Set2	Mean	Set1	Set2	Mean	Set1	Set2	Mean
k_{dm}	-0.550	-0.549	-0.550	-0.533	-0.534	-0.534	-0.895	-0.894	-0.895
k_{sm19}	0.220	0.209	0.215	0.215	0.204	0.210	0.624	0.614	0.619
k_{sm17}	0.152	0.173	0.163	0.149	0.169	0.159	0.487	0.540	0.514
k_{sm11}	0.133	0.112	0.123	0.131	0.108	0.120	0.442	0.380	0.411
k_{sm15}	0.014	0.016	0.015	0.014	0.015	0.015	0.060	0.060	0.060
k_{sm9}	0.012	0.009	0.011	0.012	0.009	0.011	0.057	0.060	0.059
k_{b7}	0.014	0.012	0.013	0.013	0.012	0.013	0.033	0.040	0.037
k_{u7}	-0.012	-0.012	-0.012	-0.013	-0.013	-0.013	-0.031	-0.033	-0.032
k_{u8}	0.006	0.001	0.004	0.006	0.002	0.004	0.023	0.017	0.020
k_{b6}	0.008	0.001	0.005	0.008	0.002	0.005	0.024	0.013	0.019
k_{u6}	-0.001	-0.000	-0.001	-0.000	-0.002	-0.001	-0.018	-0.015	-0.017
k_{b9}	-0.009	-0.002	-0.006	-0.009	-0.002	-0.006	-0.018	-0.013	-0.016
k_{b8}	-0.002	-0.001	-0.002	-0.002	-0.001	-0.002	-0.018	-0.008	-0.013
k_{u9}	0.007	0.002	0.005	0.006	0.002	0.004	0.017	0.007	0.012
k_{sm13}	0.006	0.002	0.004	0.004	0.002	0.003	0.007	0.007	0.007
